# Dread and the Disvalue of Future Pain

**DOI:** 10.1371/journal.pcbi.1003335

**Published:** 2013-11-21

**Authors:** Giles W. Story, Ivaylo Vlaev, Ben Seymour, Joel S. Winston, Ara Darzi, Raymond J. Dolan

**Affiliations:** 1 Centre for Health Policy, Institute of Global Health Innovation, Imperial College London, London, United Kingdom; 2 Wellcome Trust Centre for Neuroimaging, University College London, London, United Kingdom; 3 Center for Information and Neural Networks, National Institute for Information and Communications Technology, Suita City, Osaka, Japan; 4 Computational and Biological Learning Lab, Department of Engineering, University of Cambridge, United Kingdom; 5 Institute of Cognitive Neuroscience, University College London, London, United Kingdom; Indiana University, United States of America

## Abstract

Standard theories of decision-making involving delayed outcomes predict that people should defer a punishment, whilst advancing a reward. In some cases, such as pain, people seem to prefer to expedite punishment, implying that its anticipation carries a cost, often conceptualized as ‘dread’. Despite empirical support for the existence of dread, whether and how it depends on prospective delay is unknown. Furthermore, it is unclear whether dread represents a stable component of value, or is modulated by biases such as framing effects. Here, we examine choices made between different numbers of painful shocks to be delivered faithfully at different time points up to 15 minutes in the future, as well as choices between hypothetical painful dental appointments at time points of up to approximately eight months in the future, to test alternative models for how future pain is disvalued. We show that future pain initially becomes increasingly aversive with increasing delay, but does so at a decreasing rate. This is consistent with a value model in which moment-by-moment dread increases up to the time of expected pain, such that dread becomes equivalent to the discounted expectation of pain. For a minority of individuals pain has maximum negative value at intermediate delay, suggesting that the dread function may itself be prospectively discounted in time. Framing an outcome as relief reduces the overall preference to expedite pain, which can be parameterized by reducing the rate of the dread-discounting function. Our data support an account of disvaluation for primary punishments such as pain, which differs fundamentally from existing models applied to financial punishments, in which dread exerts a powerful but time-dependent influence over choice.

## Author Summary

People often prefer to ‘get pain out of the way’, treating pain in the future as more significant than pain now. One explanation, termed ‘dread’, is that anticipating pain is unpleasant or disadvantageous, rather like pain itself. Human brain imaging studies support the existence of dread, though it is unknown whether and how dread depends on the timing of future pain. We address this question by offering people decisions between moderately painful stimuli, and separately between imagined painful dental appointments occurring at different time points in the future, and use their choices to estimate dread. We show that future pain initially becomes more unpleasant when it is delayed, but as pain is moved further into the future, the effect of delay decreases. This is consistent with dread increasing as anticipated pain draws nearer, which is then combined with a general (and opposing) tendency to down-weight the significance of future events. We also show that dread can be attenuated by describing pain in terms of relief from an imagined even more severe pain. These observations reveal important principles about how people estimate the value of anticipated pain – relevant to a diverse range of human emotion and behavior.

## Introduction

When faced with the choice of whether to have a painful medical or dental procedure right now or in the future, many people opt to ‘get it out of the way now’. This tendency to expedite rather than delay future pain seems to challenge the generality of standard discounting models of inter-temporal choice [1]–[6]. It also suggests a fundamental principle of human valuation likely to be important for our understanding of pain and a range of health behaviors [7]–[12]. The general phenomenon is typically referred to as ‘negative time preference’ and is well replicated under controlled conditions [13]–[19]. A putative explanation is that the anticipation of primary punishments is itself inherently aversive, referred to as ‘dread’ [18]–[22]. However, the way in which dread is constructed as a function of both time and the aversiveness of outcomes is not well understood. An additional unknown property of dread is its stability in the face of biases, such as framing effects. In particular, if dread is re-framed as relief from an imagined higher amount of pain it might be possible to reduce or even reverse negative time preference. In theory if framing could eliminate dread preferences might revert to those predicted by temporal discounting alone.

A simple account proposes that, when anticipating pain, people treat each prospective unit of time as equally aversive. Here the total dread of pain accumulates linearly with increasing delay, such that the prospect of even minor pain ought to become unbearable at a sufficiently long delay. Under an alternative account, moment-by-moment dread increases as expected pain is approached in time. Under this account prospective pain is increasingly aversive with increasing delay, though at a decreasing rate. A further possibility is that moment-by-moment dread is itself prospectively discounted in time [19]. In particular, this predicts that prospective pain has a future point at which it is maximally aversive, being preferred both sooner or later. Thus we might prefer to have a dental procedure now as opposed to next week, but also next year as opposed to next week. Within the context of experimentally accessible choices, these differing accounts make testable predictions for the shape of the (dis)value function that relates prospective pain to time.

**Figure 1:**
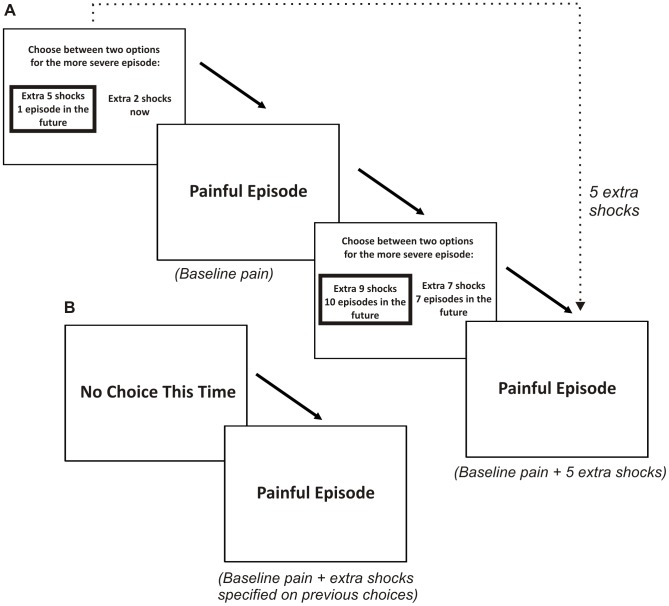
Trial structure of the task in Experiment 1. **A:** sequence of two Choice Trials, demonstrating the display of outcome options and outcome phases. The dotted arrow denotes how choices on previous trials determine expected shock rates on the future trials referred to by those choices. **B:** An example No Choice Trial.

To test these alternative models and the influence of framing effects, we examined intertemporal choice over experienced painful outcomes at different delays ranging from seconds to around 15 minutes (Experiment 1: Figure 1). The outcomes consisted of trains of brief moderately painful cutaneous electric shock stimuli delivered to the dorsum of the hand. A total of 35 participants made binary choices between shock trains with different expected shock rates (expressed in terms of the number of shocks per episode) which occurred at different points in time, where the unit of time was a single trial. Chosen outcomes were delivered faithfully at the relevant future time points. In order to achieve longer delays, choices and outcomes were interleaved, such that each choice was followed by a painful outcome, the shock rate of which was determined by choices made earlier in the experimental run. We collected intertemporal choice data in two blocks: a block in which outcomes were framed as an increase in shock rate from an expected baseline, referred to as the *pain* frame and an otherwise identical experimental block in which outcomes were framed as a decrease in shock rate from an expected maximum, referred to as the *relief* frame. In addition we examined intertemporal choices from 30 participants over hypothetical dental appointments with varying degrees of dental pain at different delays ranging from today to around 8 months (Experiment 2).

We show at a group level, for both laboratory and hypothetical outcomes, prospective aversion increases with increasing delay to the delivery of pain, but does so at a decreasing rate, consistent with a value model in which instantaneous dread increases exponentially up to the time of expected pain, allowing dread to be considered as equivalent to the discounted expectation of pain. For a minority of individuals the prospect of future pain is maximally aversive at intermediate intervals, consistent with an exponential dread function being itself prospectively discounted in time when making decisions. Framing outcomes as relief from pain attenuated, but did not reverse, overall negative preference, an effect which was best parameterized by reducing the discount rate governing the expectation of pain.

## Results

### Experiment 1

#### Participant inclusion criteria

Of the 35 participants, 2 participants were excluded from the analysis after they reported during the experiment that they did not find the shock stimuli aversive. A further 8 deterministically chose sooner pain, irrespective of the shock rate: these ‘maximum dreaders’ were excluded from the modeling analysis, since the shape of their preferences could not be reliably assessed using the experimental choices offered (a larger difference in sooner and later shock rates than those used here would be required in order to encounter indifference points for these participants).

#### Group level time preference

At the group level, participants showed a strong preference for sooner pain, at the expense of an increased number of shocks, confirming the existence of a strong effect of dread in the experiment. Overall time preference in the experiment is given by the mean probability across all choices of choosing later shocks (S2) over sooner shocks (S1), referred to as *p*(Choose S2). Since there are equal numbers of trials in which S1>S2 as in which S2>S1, overall negative time preference is indicated by *p*(Choose S2) <0.5. Group mean *p*(Choose S2) averaged across both frames and all delay lengths was significantly less than 0.5, [mean *p*(Choose S2) = 0.29, S.E. = 0.04, *N = *33, One sample *t*(32) = −5.23, *p*<0.001], this was confirmed with non-parametric testing [median *p*(Choose S2) = 0.34, One sample Wilcoxon Signed Rank test p<0.001], indicating overall negative time preference. As a result, participants chose the larger pain on 32.6% (S.E. = 3.21) of choices overall.

#### Dependence of group level time preference on delay

**Figure 2:**
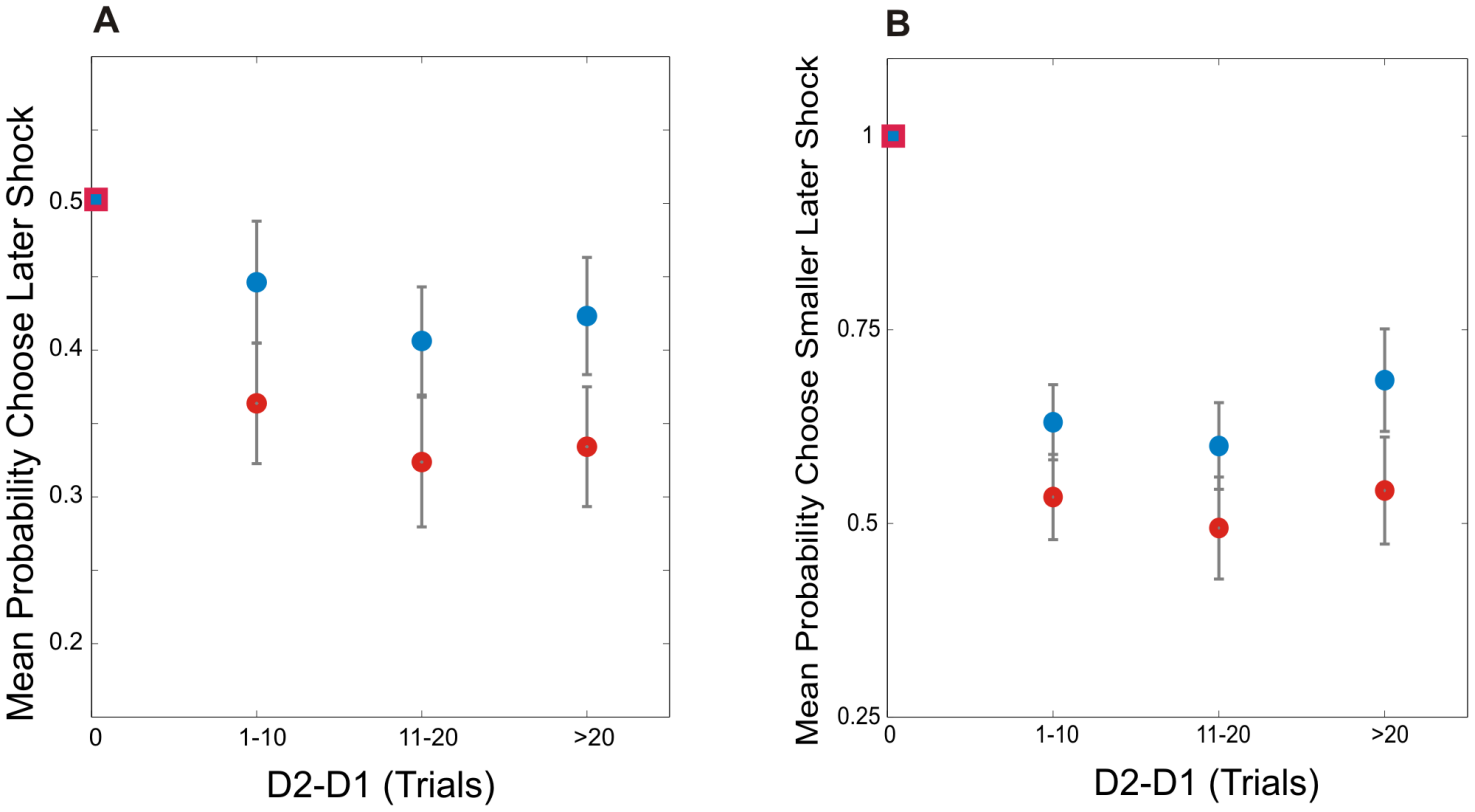
Observed time preference: Experiment 1. Mean probability across participants (*N* = 25) of choosing the more delayed shock outcome (S2) over the sooner shock outcome (S1) [referred to as *p*(Choose S2)] as a function of difference in delay between delivery of S2 and S1 (D2 – D1), expressed in units of trials. Delay difference (D2 - D1) is binned into tertiles, corresponding to short (1–10 trials), medium (11–20 trials) and long (>20 trials) delay differences. **A:** choice probabilities for all choices. At delay difference of zero, S1 and S2 would occur at the same time-point; since there are equal numbers of trials in which S1>S2 as in which S2>S1, this plot is theoretically bounded to cross the probability axis at 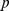
(Choose S2) = 0.5, represented by the blue and red square. Blue circles represent choice probabilities for the relief frame, red circles choice probabilities for the pain frame. Error bars represent one standard error from the between subject mean. A 2-way repeated ANOVA revealed a significant main effect of both frame [*F*(1,24) = 9.505; *p* = 0.005)] and delay [*F*(3,72) = 8.156; *p* = 0.002)], as well as a significant delay by frame interaction [*F*(3,72) = 4.169; *p* = 0.023)]. **B:** Choice probabilities for choices in which the more delayed option was a smaller number of shocks. At delay difference of zero, S1 and S2 would occur at the same time-point, under which circumstance it might be assumed that participants would show preference for the smaller number of shocks, denoted by the blue and red square.

Figure 2 shows mean *p*(Choose S2) on the two frames across the 25 subjects included in the modeling analysis, as a function of delay length, where the latter is expressed as the difference in delay between the two choice options. Since there are equal numbers of trials in which S1>S2 as in which S2>S1, and option presentation is counterbalanced, *p*(Choose S2) at a delay difference of zero is theoretically bounded at 0.5. Delay difference (D2-D1) is binned into tertiles, corresponding to short (1–10 trials), medium (11–20 trials) and long (>20 trials) delay differences. A 2-way repeated measures analysis of variance (ANOVA) revealed a significant main effect of frame [*F*(1,24) = 9.5 *p* = 0.005)], whereby participants chose sooner shocks less frequently in the *relief* frame. There was also a significant main effect of delay [*F*(3,72) = 8.2; *p* = 0.002)], as well as a delay by frame interaction [*F*(3,72) = 4.2; *p* = 0.023)]. Non-parametric pair-wise comparisons between zero, short, medium and long delay differences (across both frames combined) revealed a significant decrease in *p*(Choose S2) between zero and short delay differences [*p*(Choose S2, Short) <0.5, Wilcoxon Signed Rank test, *p* = 0.004] and between short and medium delay differences (Wilcoxon Signed Rank test, *p* = 0.014), but no significant change between medium and long delay differences (Wilcoxon Signed Rank test, *p* = 0.398), suggesting that negative time preference decreases at longer delay differences, rather than being constant. In particular the *slope* of the dependence of *p*(Choose S2) on delay provides a proxy for the rate of time preference. We therefore tested the hypothesis of decreasing negative time preference by performing a further 2-way repeated measures ANOVA, entering the slope of *p*(Choose S2) between each category of delay difference as the dependent variable. This second ANOVA demonstrated a significant main effect of frame on the rate of time preference [*F*(1,24) = 15.5; *p* = 0.001)], as well as a significant main effect of delay [*F*(2,48) = 4.4; *p* = 0.033)], thus rejecting the null hypothesis of constant negative time preference. There was no significant delay by frame interaction in this analysis [*F*(2,48) = 1.6; *p* = 0.205)]. Non-parametric pairwise comparisons revealed that the effect of delay was driven by a significantly more negative slope between short and medium delays than between medium and long delays (Wilcoxon Signed Rank test, *p* = 0.013), consistent with diminishing negative time preference. This suggests the rate of accumulation of dread diminishes with increasing delay, suggesting that on average instantaneous dread increases in time, rather than being constant.

#### Classification of participants by individual time preference

**Figure 3:**
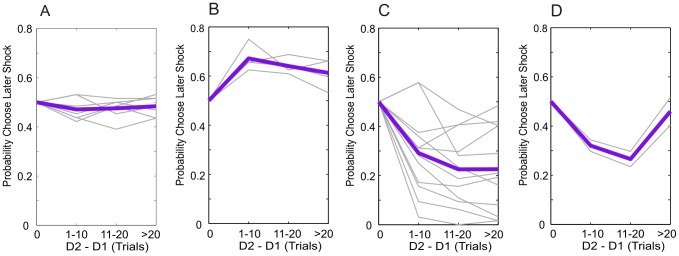
Observed time preference in individual participants categorized by time preference. *p*(Choose S2) as a function of delay difference, expressed in units of trials, for all 25 participants included in the modeling analysis. Choice probabilities shown are the mean of those on the two frames. Delay difference scaling is identical that in Figure 2. Time preference is approximated by the slope of the choice probability lines. **A**: participants with no significant time preference at any delay. **B**: participants who show positive time preference, but no significant negative time preference at any delay. **C**: participants who show negative time preference, but no significant positive time preference at any delay. **D**: participants with initial negative time preference followed by significant positive time preference at longer delays. Data are plotted as solid lines to assist visualization of the choice patterns. Each gray line represents data from a single participant. The bold purple lines represent the between-subject means in each category. At delay difference of zero, S1 and S2 would occur at the same time-point; since there are equal numbers of trials in which S1>S2 as in which S2>S1, the plots are theoretically bounded to cross the probability axis at *p*(Choose S2) = 0.5.

The group level data displayed in Figure 2 conceal substantial heterogeneity in the response patterns of individual participants. We therefore categorized the 25 participants whose data contributes to Figure 2 according to their individual pattern of time preference. We identified four mutually exclusive categories: *zero* time preference, *positive* time preference, *negative* time preference, and *reversing* time preference (Figure 3). Participants were classified as having *zero* time preference if *p*(Choose S2) showed no significant deflections from 0.5 at any delay difference (Binomial test, *α = *0.05) (7/25, Figure 3A). The zero time preference group chose the option with the smaller shock rate on 88% of choices [mean *p*(Choose Smaller) = 0.88, SE = 2.8], demonstrating that this group did not simply respond randomly, but tended to choose the less painful stimulus, irrespective of delay. Participants were classified as having *positive* time preference if they displayed significant increases in *p*(Choose S2), but no significant decreases (Fisher Exact test, *α = *0.05) (4/25, Figure 3B), as having *negative* time preference if they displayed significant decreases in *p*(Choose S2), but no significant increases (12/25, Figure 3C), and as having *reversing* time preference if they displayed significant increases in *p*(Choose S2), as well as significant decreases (2/25, Figure 3D). The two participants with *reversing* time preference both displayed initial negative, followed by positive time preference, a pattern that would be consistent with prospective dread being itself discounted in time.

#### Group level modeling analysis

**Figure 4:**
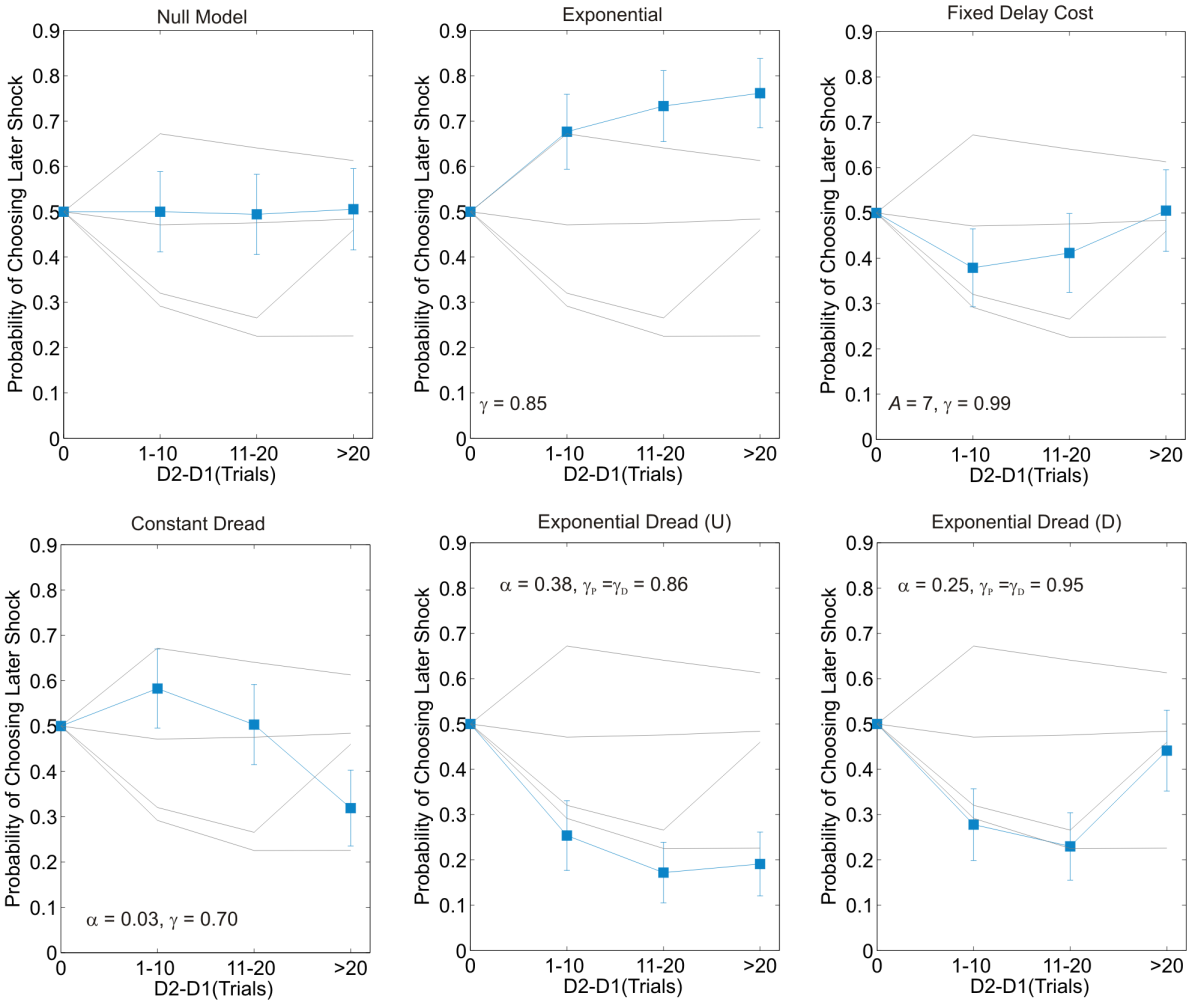
Model predictions on the task: Experiment 1. *p*(Choose S2) as a function of delay difference according to alternative models of dread. Choice probabilities shown are the mean of those on the two frames. Delay difference scaling is identical that in Figure 2. The fine gray lines represent mean *p*(choose S2) for the four participant subgroups shown in Figure 3. Data points marked by blue squares, joined with lines for illustrative purposes, represent model data simulated at the parameter values denoted in each panel. These do not represent the results of model fitting, but serve to illustrate the basic form of the alternative model predictions. Notably different parameterizations of the more complex models can produce diverse shapes of choice frequency plot (see Figures S2, S3, S4, S5). Error bars represent one standard deviation of the binomial distribution. In each case the softmax inverse temperature parameter, 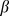, is set to 0.25, a representative value.

We compared alternative accounts for the computations underlying the observed patterns of time preference by fitting a series of dread-discounting models of increasing complexity. Each model parameterized the function by which future pain was disvalued, and therefore the value of each of the binary choice options presented. Values were transformed into predicted probabilities of choosing either option according to a softmax activation function (see Methods). In order to draw comparisons between models with differing architecture and complexity, we calculated the Bayesian Information Criterion (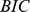) for each model at the group level, according to a fixed-effects scheme, by summing the 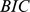 values for model fits to individual data. The 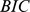 favors models with higher likelihood estimates and penalizes increasing model complexity, where lower values of 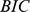 indicate a more favorable model fit. Figure 4 displays choice probabilities on the paradigm itself predicted by representative parameterizations of each model (blue lines), as well as the mean choice probabilities from each of the four participant sub-groups. (Notably the shape is also dependent on the parameters of each model and the parameter dependence of the more complex models is illustrated in Figures S1, S2, S3, S4, S5).

Each model shares the same general form, but differs in the manner in which dread varies as a function of time and outcome magnitude. In all models, we assume that the total aversive value of a prospective option (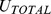) is equal to the aversive value associated with accumulated sum of future dread up until the time (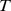) of the actual pain plus the aversive value related to the pain itself:
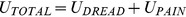
(1)In all models, we also assume exponential discounting of pain, where 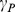 represents the discount factor applied to the future pain, and 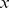 represents the shock rate. Thus:
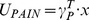
(2)Hence what differs between the models is the way in which 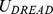 depends on 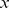 and *T*. Although different forms of discounting function, such as hyperbolic and quasi-hyperbolic, are of importance in standard models of financial discounting, they have a relatively subtle effect here, since the more complex functional forms resulting from the addition of dread depend little on the precise shape of the basic discounting function; we therefore adopt exponential discounting for the sake of simplicity.

In the following analysis 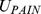 is assumed to be a linear function of the shock rate, *x*. Since there was no *a priori* reason for this assumption, besides parsimony, we performed an identical model comparison in which (negative) 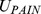 was a concave function of the shock rate. The parameters of this concave utility function were derived empirically, separately for each subject, and outside of the main model fitting procedure, by fitting a three-parameter Weibull function to participants' subjective ratings of stimuli with differing shock rates (shown in Figure S6).

*Model 1:* The first model, the Null model, assumes that the prospective disvalue of pain depends only on the stated number of shocks, and not on the delay, i.e. 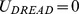 and 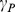 = 1 such that:
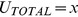
(3)As shown in Figure 4A, this model predicted a net 50% probability of choosing later shocks on our choice paradigm. This arose of the fact that on half the presented options the later outcome carried a larger number of shocks and on half the options the later outcome carried a smaller number. The Null model did not adequately capture observed choice patterns at the group level (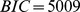).

*Model 2:* The second model, the Exponential Discount model, extends the Null model to add an exponential discount rate for pain, 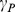, but again assumes that 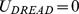, such that:
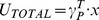
(4)The Exponential Discount model predicted overall preference for later shock (Figure 4). This model improved the group-level likelihood by comparison with the Null model, consistent with some participants demonstrating positive time preference however this was not sufficient to compensate for an increase in complexity over the Null model (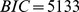).

*Model 3:* The third model, which is the simplest model to incorporate dread, assumes a constant benefit or cost from anticipation accruing from any delayed outcome, which does not scale with the size of the outcome or the delay to its delivery. In other words, outcomes accrue a fixed cost from anticipation.
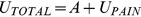
(5)Where 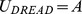 is the fixed constant of dread, assumed to be negative. Despite the additional parameters, this Fixed Delay Cost model substantially outperformed both the Null and Exponential Discount models (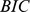 improvements of 368 and 492 respectively).

**Figure 5:**
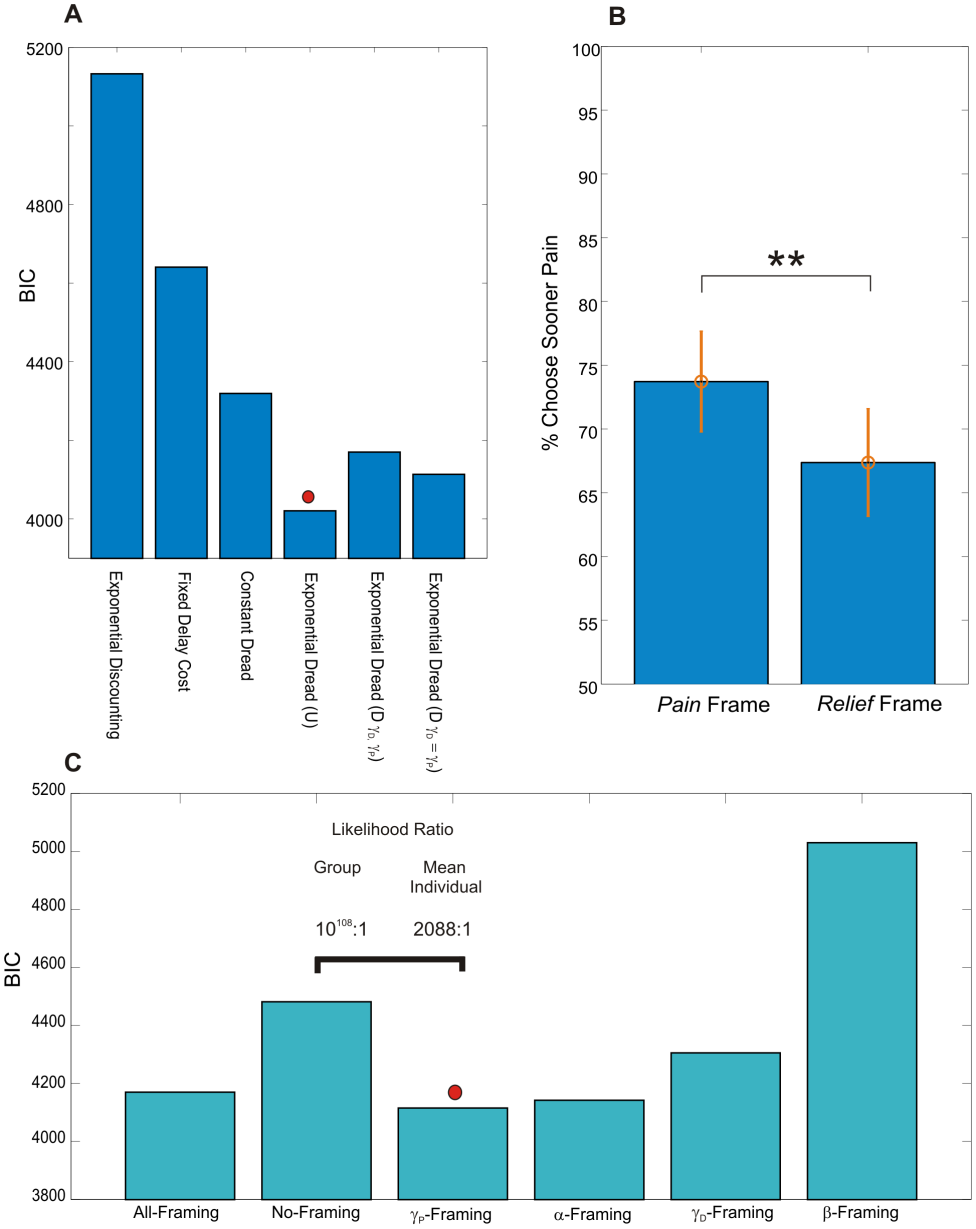
Model comparison and framing effects: Experiment 1. **A**: Bayesian Information Criterion (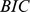), summed across participants (*N* = 25) for the alternative models. Lower values of 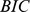 indicate better fits of the model. Exponential Dread outperformed other models, with Undiscounted Exponential Dread providing the most parsimonious fit at the group level, indicated by the red circle. **B**: Mean frequency of choosing sooner pain across all choices by all participants in either frame. Error bars show one standard error from the mean in each direction. Two-tailed paired t-test showed significant difference between the two frames *t*(32) = 2.84, *p* = 0.0077. This result was confirmed with non-parametric testing for differences between paired samples using the Wilcoxon Signed Rank test, which revealed significant differences between the two medians (*N* = 33, *Z* = −2.6, *p* = 0.0093). **C**: Results of fitting the general form Exponential Dread model to both pain and relief frames, whilst restricting which parameters were allowed to vary between frames. In the unrestricted framing model (All-Framing) all four parameters, the inverse softmax temperature, 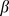, the discount parameters, 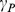 and 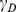, and the anticipation parameter, 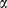, were applied separately to each frame, yielding an eight parameter model. In the fully restricted framing model (No-Framing) all parameters were constrained to be equal across frames, yielding a four-parameter model. The best fit, indicated by the red circle, was provided by a four-parameter model in which 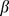, 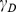 and 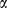 were fixed across frames, leaving between-frame differences explained by differences in 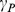 (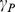-Framing). Likelihood ratios are displayed at both the group level and the individual level, strongly favoring the 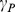-Framing model over the No-Framing model at both the group (fixed effects) (LR = 10^108^∶1, χ2 = 497.3, *p*<0.001, *d. f.* = 25) and individual levels (Mean individual LR = 2088∶1, χ2 = 19.9, *p*<0.001, *d. f.* = 1) .

*Model 4:* The fourth model, Constant Dread, assumes that dread is constant over time, where 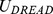, is given by the prospective sum of dread across the delay:
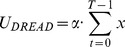
(6)Hence dread accumulates linearly when pain is viewed from the perspective of an increasing delay. As in all dread-discounting models discussed here, total dread is added to the discounted value of the delayed pain itself in order to compute the overall disvalue of delayed pain. Dread is weighted in this sum by the parameter 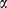, equivalent in this case to a proportionality constant for the linear increase in dread. Figure S2 outlines the parameter dependence of the Constant Dread model. The time dependence of total dread embodied by this model substantially improved the model fit compared with Fixed Delay Cost (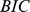 improvement of 321, see Figure 5A).

*Model 5:* The fifth model, Exponential Dread, assumes an increasing time-course of instantaneous dread, such that dread increases exponentially until the actual time of pain. Parsimoniously, the model assumes that the exponential rate governing the increase in dread is identical to the rate by which pain is discounted, 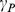, such that dread becomes simply equivalent to the predicted (discounted) value of future pain. In addition, the general form of the Exponential Dread model allows the exponential rise of dread to be itself discounted in time by a further discount factor, 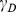, such that:
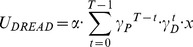
(7)


Figure S2 outlines the parameter dependence of the general form Exponential Dread model. We tested this general form, as well as two nested variants. The first variant, which we term Undiscounted Exponential Dread assumes that dread is not itself prospectively discounted, such that 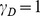:
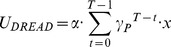
(8)


Figure S3 outlines the parameter dependence of this Undiscounted Exponential Dread model, which predicts that preference for sooner shock saturates at longer delays. The second variant, Restricted Discounted Exponential Dread, assumes that the rate of exponential rise of dread is the same as the rate of exponential discounting of dread, such that 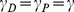, giving:
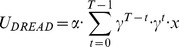
(9)


Which further simplifies as follows:
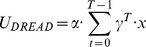
(10)

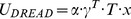
(11)


All forms of the Exponential Dread model are capable of predicting diminishing negative time preference. Furthermore, if dread itself is prospectively discounted (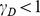), reversals of time preference can occur, such that prospective pain is maximally aversive at intermediate delays (Figures S2 and S3).

Consistent with the behavioral observation of diminishing negative time preference, (Figure 2) Exponential Dread models were the best performing at the group level (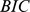 improvement of 149 over Constant Dread for general form Exponential Dread). The restricted variants were favored over the general form, with Undiscounted Exponential Dread providing the most parsimonious fit out of all models tested at the group level (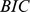 improvement of 150 over the general form), followed by Restricted Discounted Exponential Dread (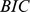 improvement of 57 over the general form).

#### Group level framing effects

Across participants, the mean frequency of choosing sooner pain in the *pain frame* was 73.7% (S.E. = 3.95) and 67.3% (S.E. = 4.22) in the *relief frame* (Figure 5B). A two-tailed paired t-test showed that this difference was significant: 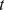
(32) = 2.84, 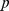 = 0.0077. The result was confirmed with non-parametric testing for differences between paired samples using the Wilcoxon Signed Rank test, which revealed significant differences between the median choice frequencies (*N* = 33, 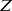 = −2.6, 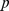 = 0.0093).

Analysis of the framing effect was extended using the general form Exponential Dread model to test which parameters of the model best accounted for the differences in intertemporal choice between *pain* and *relief* frames. Thus, we performed a second model comparison whilst restricting which parameters were allowed to vary between frames. The general form model was chosen for this analysis in order that predictions regarding the basis of the framing effects were not dependent on accepting a specific version of the model. The results of model comparison across frames are shown in Figure 5C.

The two models with the lowest 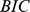 were the model in which 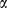 is allowed to be free (i.e. the discount factors 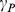 and 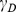 and the choice temperature 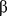 are fixed; 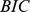 = 4142), and the model in which 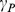 is allowed to be free (i.e. the dread discount factors 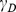, the dread weighting parameter, 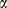, and the choice temperature 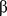 are fixed; 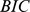 = 4115), with a 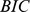 difference of 27, indicating substantial support for the 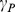-framing model. These results suggest that the framing effect was most parsimoniously accounted for by changes in the discount rate governing the rate of increase in the dread of future pain, 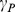. Likelihood ratio tests rejected the (null) No-Framing model in favour of the 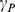-Framing model at both the group (fixed effects) (LR = 10^108^∶1, χ2 = 497.3, *p*<0.001, *d. f.* = 25) and individual (Mean individual LR = 2088∶1, χ2 = 19.9, *p*<0.001, *d. f.* = 1) levels.

#### Modeling with non-linear utility of pain

**Figure 6:**
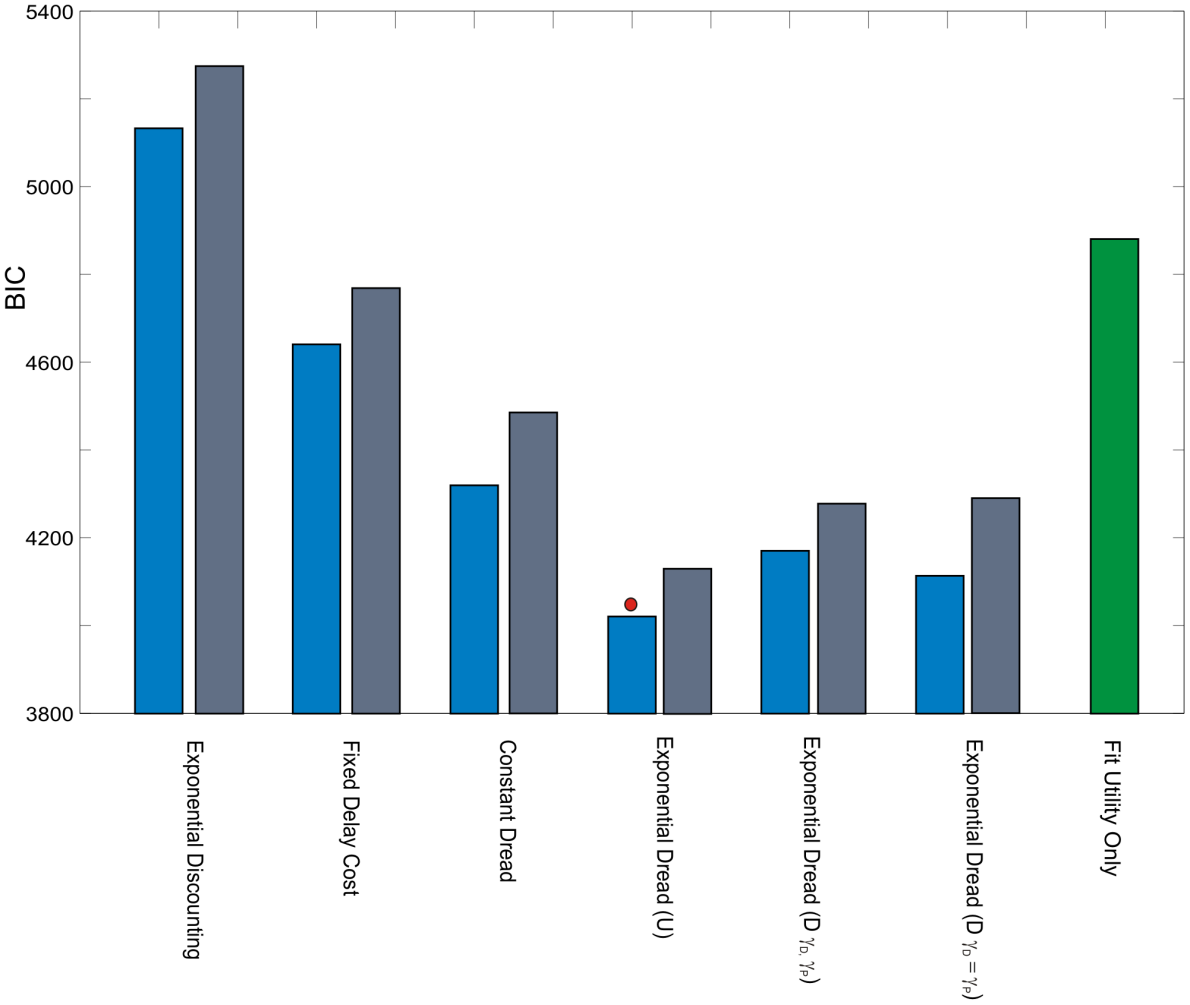
Model comparison with non-linear utility: Experiment 1. The results of an identical model comparison, performed with subject-specific non-linear utility functions for pain, derived from subjective ratings of stimuli with differing shock rates, as shown in Figure S6. Blue bars represent summed 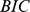 values for linear utility models, gray bars the 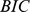 values for non-linear utility models. For each alternative model using linear utility provided better model fits, as indicated by lower 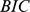 values. Importantly, the rank order of the models was largely unchanged using non-linear utility, the only exception being that the general form Exponential Dread model outperformed the Restricted Discounted version with non-linear utility, but not with linear utility. The green bar labelled “Fit Utility Only” represents the result of implementing the Null model with a freely fitted three-parameter Weibull utility function, showing that a variable utility function alone was unable to account competitively for the observed data.

Within the set of experimental choices offered, absolute shock rate is weakly correlated with the delay difference (Pearson *r* = 0.12) between the two options. As a result, it is possible that some of the variance in the data could be accounted for by the shape of the utility function for pain, independent of the effect of delay. To examine the contribution of variable utility, we fitted a version of the Null model in which the pain utility function (implemented as a three-parameter Weibull function) was allowed to freely vary between subjects. This model (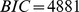) illustrated that the utility function alone was unable to account for the full range of the observed findings. We performed further model comparison of the dread-discounting models under a concave utility function for pain. The rank ordering of the model fits was unchanged under concave utility, whilst the overall quality of fits was higher with linear utility (Figure 6), demonstrating that the results remain robust to changes in the utility function of pain. We could speculate from these results that subjects, having only sampled the extremes of shock rate prior to taking part in the experiment, did not have precise insight into the shape of their own utility functions for shock rate, and may have therefore used linear utility as a heuristic.

#### Modeling analysis of sub-groups

**Figure 7:**
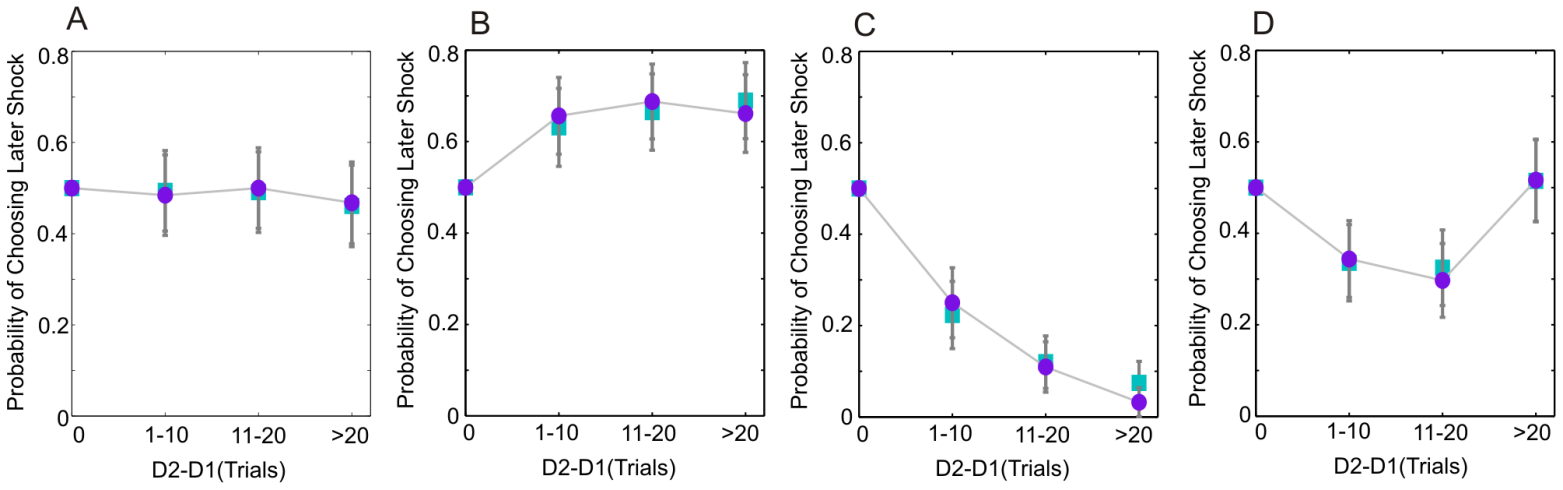
Time preference of sample participants on Experiment 1 and fits of the (discounted) Exponential Dread model. Observed *p*(Choose S2), combined across both frames, as a function of delay difference, expressed in units of trials, is displayed for a single participant from each of the four subgroups shown in Figure 3, indicated by the purple circles. Delay difference scaling is identical that in Figure 2. Data simulated from the general form Exponential Dread model at the maximum likelihood parameter estimates for each participant, subsequently combined across frames, are plotted as cyan squares. Error bars represent one standard deviation of the binomial distribution. **A**: a participant with *zero* time preference **B**: a participant with *positive* time preference (left hand column). **C**: a participant with *negative* time. **D**: a participant with *reversing* time preference: showing initial negative time preference reverting to positive time preference at longer delay differences. The general form of the Exponential Dread model adequately captures all four patterns of time preference.

At the sub-group level, we hypothesized that the Null model would perform best in the *zero* time preference group, that Exponential Discounting would perform best in the *positive* time preference group, that Undiscounted Exponential Dread would perform best in the *negative* time preference group and that either general form or Restricted Discounted Exponential Dread would perform best in the *reversal* group. In the *zero* time preference group, the two models with the lowest 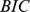 estimates were indeed the Null model (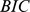 = 755) and Undiscounted Exponential Dread (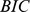 = 793), suggesting evidence in favor of the Null model in this group. In the *positive* time preference group the two models with the lowest BIC estimates were Fixed Delay Cost (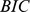 = 961) and Exponential Discounting (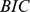 = 964), with a 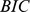 difference of 3.5, unexpectedly providing weak evidence in favor of the Fixed Delay Cost model in this group. Closer inspection revealed that the improved fit of this model was driven by a single participant, who displayed a degree of negative time preference on the *pain* frame. As expected, in the negative time preference group the best performing model was Undiscounted Exponential Dread (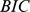 = 1868), followed by Restricted Discounted Exponential dread (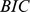 = 1941). Also as expected, in the reversal group, the best performing model was Restricted Discounted Exponential dread (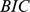 = 370), followed by the general form Discounted Exponential Dread (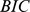 = 372) (followed by Undiscounted Exponential Dread, 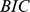 = 374), indicating evidence in favor of dread being discounted in this sub-group. Notably, alternative parameterizations of the general form Exponential Dread model are able to account for the patterns of time preference displayed by each of the four sub-groups, as shown in Figure 7, which presents data from a single participant within each group, along with the maximum likelihood estimates predicted by the general form Exponential Dread model.

The 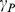-framing model provided the most parsimonious account of between-frame differences for all participant sub-groups. Framing effects were most prominent in the negative and reversing time preference groups, which together accounted for all participants with significant behavioral framing effects in the expected direction (9 participants, Fisher exact test *p*<0.05). Consistent with this, 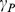 was significantly higher in the pain frame (less discounting of pain, faster accumulation of dread) than in the relief frame in the *negative* time preference group [Wilcoxon signed rank test, *d. f.* = 14, *p* = 0.006]. No subjects in the *zero* time preference group showed significant framing effects, and there were no significant between-frame differences in 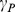 in the zero time preference sub-group [Wilcoxon signed rank test, *d. f.* = 11, *p* = 0.57]. A single subject in the *positive* time preference group showed a significant behavioral framing effect in the reverse direction (higher discounting in the *pain* frame), in this case parameterized as a lower 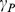 in the pain frame, suggesting that the direction of the framing effect described here relies on subjects displaying a significant degree of dread.

### Experiment 2

#### Participant inclusion criteria

All 30 participants were included in the analysis.

#### Group level time preference

In Experiment 2 participants made 70 choices between two possible timings for a hypothetical dental appointment. They were informed that the appointment would last for 15 minutes and that the experience would be painful. Participants were also informed that the appointment was routine and that the timing would not affect their dental health. They were asked to imagine that the dental surgery was situated close to their home, such that they could attend an appointment almost immediately should they so wish. The severity of the pain for each possible appointment was described as a percentage, where 100% represented the worst imaginable dental pain. Since outcome magnitude was described in experiential terms, a linear utility function was assumed over percentage pain intensities. The appointment delays ranged from “today” to 237 days in the future, and were described in units of days. As for Experiment 1, there were an equal number of choices for which the larger magnitude pain was the sooner option as choices in which the (identical) larger magnitude pain was the later option, therefore the probability of choosing the later option, in this case *p*(Choose A2) where A2 refers to Appointment 2, reflects time preference in the same manner as for Experiment 1.

Consistent with Experiment 1, at the group level, participants showed a strong preference for sooner dental appointments, at the expense of more severe dental pain: group mean *p*(Choose A2) averaged across all delay lengths was significantly less than 0.5, [mean *p*(choose A2) = 0.38, *N* = 30, SE = 0.025, One sample *t*(29) = −4.56, *p*<0.001], a result which was confirmed with non-parametric testing [median *p*(Choose A2) = 0.39, One sample Wilcoxon Signed Rank test p<0.001], indicating overall negative time preference.

#### Dependence of group level time preference on delay

**Figure 8:**
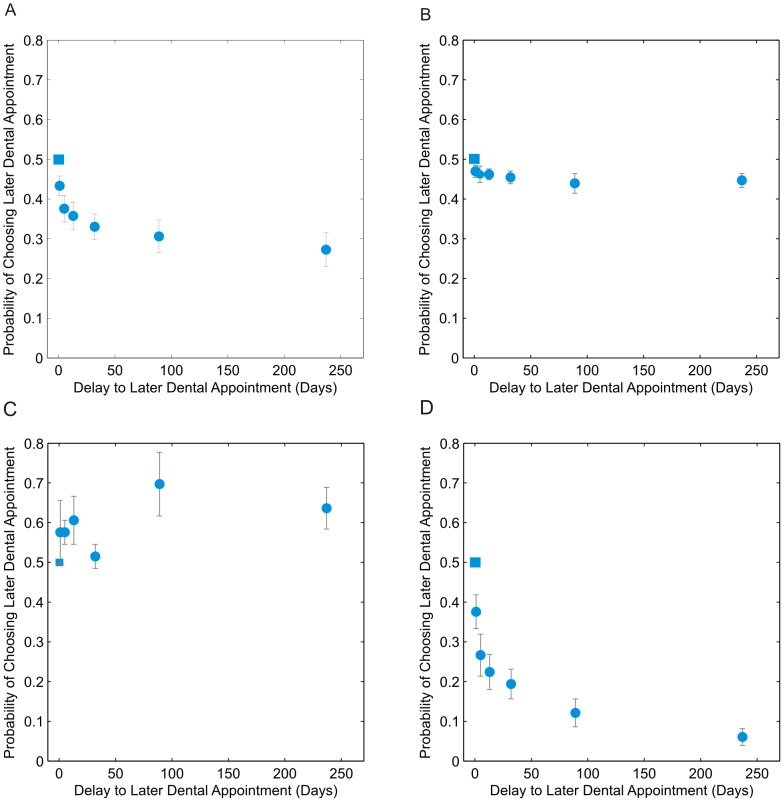
Time preference for a hypothetical painful dental appointment: Experiment 2. Observed *p*(Choose A2) is plotted as a function of the delay to the later appointment; the sooner appointment was always at 0 days, i.e. “today”. Error bars represent one standard error from the group mean. **A**: group mean *p*(Choose A2) for all participants (*N = *30). **B**: mean *p*(Choose A2) in the zero time preference group (*N = *12). **C**: mean *p*(Choose A2) in the positive time preference group (*N = *3). **D**: mean *p*(Choose A2) in the negative time preference group (*N = *15).

To assess the dependence of time preference on delay at the behavioral level, and to facilitate comparison with Experiment 1, delays to the later dental appointment were grouped into short (1–5 days), medium (13–32 days) and long (89–237 days) categories. A one-way analysis of variance (ANOVA) revealed a significant main effect of delay [*F*(3,116) = 8.1 *p*<0.001)]. Non-parametric pair-wise comparisons between zero, short, medium and long delays revealed a significant decrease in *p*(Choose A2) between zero and short delay differences [*p*(Choose A2) <0.5, Wilcoxon Signed Rank test, *p*<0.001], between short and medium delay differences (Wilcoxon Signed Rank test, *p* = 0.0025), and between medium and long delay differences (Wilcoxon Signed Rank test, *p* = 0.022), suggesting consistent negative time preference at the group level. Figure 8A displays the group mean *p*(Choose A2) for Experiment 2 at all delay lengths offered. The data reflect the finding of consistent negative time preference. Diminishing negative time preference with increasing delay can be clearly appreciated from the shape of the plot.

#### Classification of participants by individual time preference

We categorized the 30 participants according to their individual pattern of time preference, using an identical method as for Experiment 1: 12 out of 30 participants showed *zero* time preference, 3 out of 30 showed *positive* time preference, and the remaining 15 out of 30 showed *negative* time preference. In this experiment, no participants displayed *reversing* time preference, i.e. none displayed significant increases in *p*(Choose A2), as well as significant decreases. Figures 8B–D display group mean *p*(Choose A2) at each delay length for the *zero*, *positive* and *negative* time preference groups respectively.

#### Group level modeling analysis

**Figure 9:**
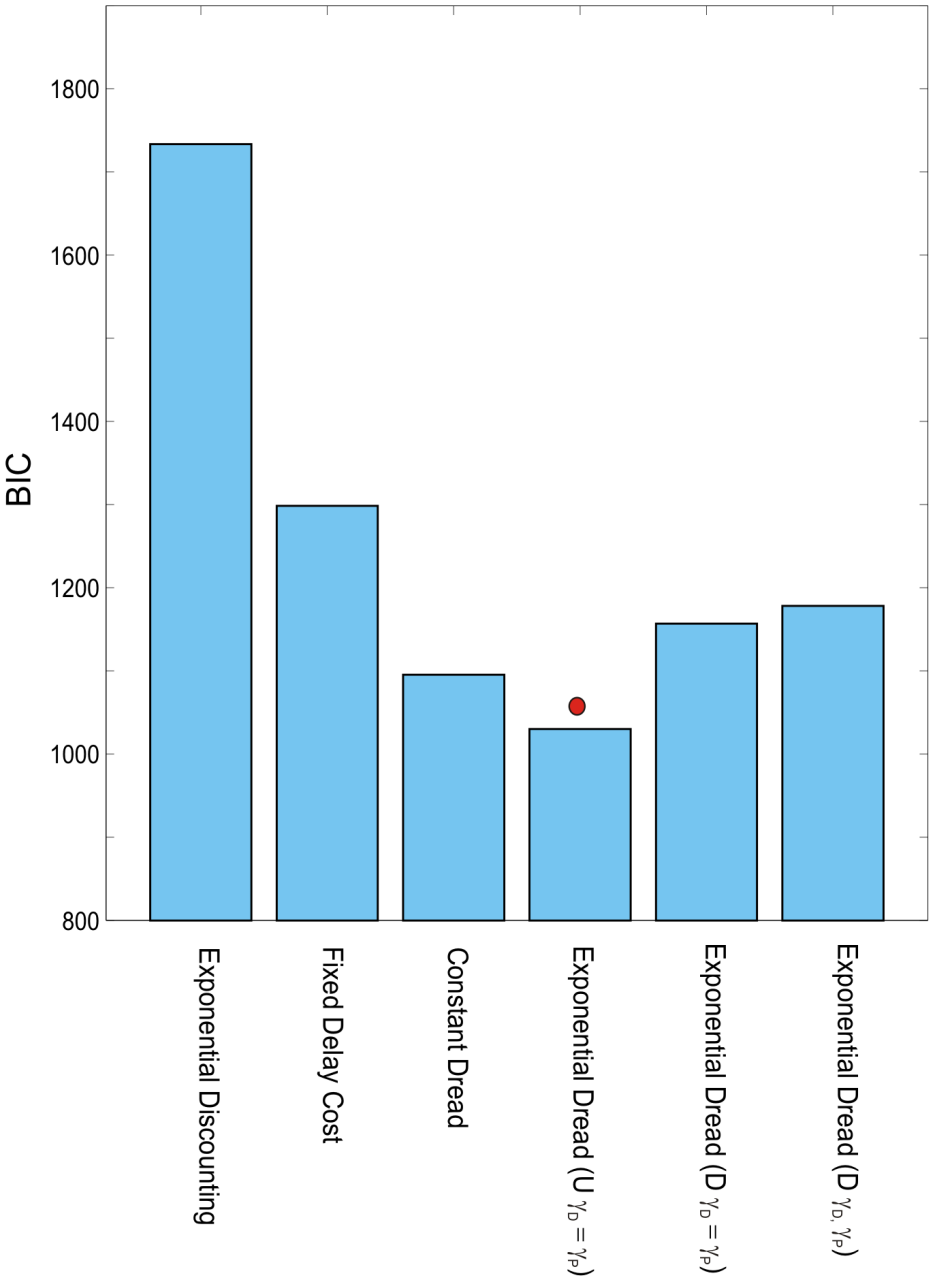
Model comparison for Experiment 2. Bayesian Information Criterion (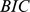), summed across participants (*N* = 30) for the alternative models. Lower values of 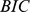 indicate better fits of the model. Undiscounted Exponential Dread provided the most parsimonious fit at the group level, indicated by the red circle.

The results of group level model comparison for Experiment 2 are shown in Figure 9. Consistent with the shape of the group level choices (Figure 8), Undiscounted Exponential Dread was the best performing model at the group level (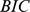 = 1036), followed by Constant Dread (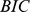 = 1115). These two models outperformed both the restricted variant of Discounted Exponential Dread (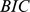 = 1162), general form Discounted Exponential Dread Constant Dread (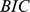 = 1179), Fixed Dread Cost (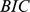 = 1418). Exponential Discounting (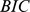 = 1784) and the Null model (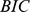 = 1791).

#### Modeling analysis of sub-groups

At the sub-group level, we hypothesized that the Null model would perform best in the *zero* time preference group, that Exponential Discounting would perform best in the *positive* time preference group and that Undiscounted Exponential Dread would perform best in the *negative* time preference group. In the *zero* time preference group, the best performing model was in fact Undiscounted Exponential Dread (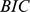 = 218), followed by the restricted form of Discounted Exponential Dread (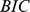 = 229), followed by the Null model (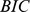 = 232). This unexpected result is consistent with the observation that participants in the *zero* time preference group displayed a small but consistent degree of negative time preference, as shown in Figure 8B. In the *positive* time preference group the two models with the lowest BIC estimates were Exponential Discounting (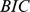 = 176) and Undiscounted Exponential Dread (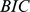 = 179), a BIC difference of 3.1, providing weak evidence in favor of Exponential Discounting in this group. Similarly, as predicted, in the *negative* time preference group the best performing model was Undiscounted Exponential Dread (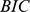 = 633), followed by Constant Dread (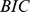 = 674).

## Discussion

We compared alternative accounts for how aversive (dis)value is constructed as a function of time using two experimental paradigms in which participants made choices between painful outcomes occurring at different delays in the future: in the first the outcomes were moderately painful electric shocks which were experienced for real, and in the second the outcomes were hypothetical painful dental appointments.

In accordance with previous studies we found that most participants (26 out of 33 in Experiment 1, see Table S1 and Text S1) exhibited dread for pain. These participants preferred to experience the same pain sooner rather than later and were willing to accept more pain in order to hasten its occurrence. The observed behavior for both real and hypothetical painful outcomes revealed that negative time preference initially increased with increasing delay, but saturated at long delay. This pattern was best accounted for by a dread-discounting model in which dread increases exponentially as pain is approached in time. The total utility from dread is then given by the prospective sum of dread, where the extent to which an individual incorporates dread can be described in terms of the weighting parameter, α. We termed this model Exponential Dread. We showed also that dread is modulated by relief framing, an effect which was captured by modulation in the rate of instantaneous dread increase.

Our findings extend those of Berns and colleagues, who compared a Constant Dread model and an Exponential Discounting model without dread in the context of choice between delayed shocks predicted by a cue [18]. In the latter study, the Constant Dread model provided better fits to both the behavioral data and the BOLD response in several regions of interest (right primary and secondary somatosensory cortices, caudal anterior cingulate cortex and right posterior insula) than the model without dread, demonstrating a neural correlate of dread. We have used behavior to probe the dependence of dread on delay, and in so doing provide direct empirical support for Exponential Dread, corresponding to the original form of the anticipation-discounting model proposed by Loewenstein [19].

We suggest that both moment-by-moment dread and the temporally discounted value of pain itself increase as pain is approached in time. This leads to a putative simplification, embodied by the Exponential Dread model, that both are one and the same signal – simply the instantaneous anticipation of pain. An increasing aversiveness by time function for the anticipation of pain bears similarity to observations in studies of fear conditioning. For example, the ability of fear to potentiate the startle response is specific to the learned time interval between conditioned stimulus (CS) and unconditioned stimulus (UCS) [23]–[26]. Thus, following the CS, fear behaviors increase to reach a maximum at the predicted time of UCS onset. Similarly, in human subjects instructed to expect shock after a stated delay, physiological measures of fear such as galvanic skin response (GSR) and heart rate both increase roughly exponentially in the period immediately preceding the predicted time of shock delivery [27]–[29]. Consequently, the anticipation of pain can be considered as resembling a temporally discounted value signal, assuming a low level when pain is distant and increasing as pain is approached. Indeed, we suggest it is possible that the overall motivational value of pain reflected across many instances of pain-related decision-making incorporates, to a varying degree, a prospective sum of this anticipation, comprising the dread term of the dread-discounting model.

A small proportion of participants (2 out of 25 in Experiment 1) exhibited negative time preference which reverted to positive time preference at longer delays, consistent with an Exponential Dread model in which dread is itself prospectively discounted. However, we acknowledge that we have insufficient evidence to support this conclusion at the group level, and we do not observe this pattern for hypothetical outcomes over the range of delays offered. The key prediction here is that the (negative) value function for pain has a maximum at an intermediate time point, as opposed to increasing or decreasing steadily across time. Such maxima would predict dynamic preference reversals for delayed aversive outcomes, whereby people would be most likely to attempt to avoid the dreaded outcome at the point of maximal aversion. We therefore speculate that high discounting of dread may contribute to avoidant psychopathology [10], [11], [30], [31]. We propose that a general form Exponential Dread model is well-placed to parameterize individual differences in the valuation of future pain, and a possible direction for future research will be to investigate the discounting of dread in clinical populations.

Whilst dread clearly represents a departure from economic theories of behavior, such as Rational Choice theory [32], [33], the dread-discounting models presented here retain assumptions of intertemporal independence. In other words the models assume that prospective dread is simply the sum of the instantaneous anticipation of future pain. This assumption is particularly relevant to the design of Experiment 1, which interleaves choices and outcomes, such that participants can be making choices about future pain, whilst currently anticipating the results of their previous choices. If participants keep track of their previous choices, dread from previous choices would overlap with the estimated dread of the current choice options. Additive independence of dread entails that previous dread simply adds the same amount to the value of both choice options and therefore is eliminated from the value of the current choice options (this is the case since the softmax activation function subtracts the value of the two choice options, see Methods: Equation 19). Whether dread from different time periods indeed simply adds together linearly in this manner forms an important question for future study.

We show that choices that expedite pain were more frequent when the same outcomes were framed as an increase in pain than when framed as a decrease in pain, a demonstration that framing biases exert a strong effect in situations associated with dread. Modeling analysis of participant subgroups indicated that the framing effect we report is only present in participants who display significant dread (Figures S8 and S9). The observation of framing may be similar to that which underlies the well-known sign effect, in where discount rates for rewards are typically different from those for punishments [34], [35]. A model in which between-frame differences in temporal value functions were determined by changes in the rate of accumulation of dread, here equivalent to the discount rate for pain, provided the most parsimonious account for these effects of framing, suggesting that differential anticipation is a sufficient explanation for the sign-effect in this context. This observation is however bound to the framework of the dread-discounting model. It is possible for example that framing induced changes in the instantaneous utility function for pain [34].

We have suggested that increasing instantaneous dread may represent a fundamental principle of anticipated aversion. A multitude of factors, which may interact with the effect of delay, are likely to influence the valuation of future pain in real-world contexts. Nevertheless we show the functional form of dread appears conserved across two very different experimental contexts: in the context of real painful outcomes experienced at delays of up to approximately 15 minutes, and in the context of an imagined painful experience at delays of up to approximately 8 months. A relevant observation here is that the form of the dread function appears to demonstrate scale invariance, as evidenced by a similar shape when making choices over delays expressed in different units of time (trials or days). As a result the magnitude of prospective dread at a given delay is likely to depend upon the psychological construal of the time scale. Scale invariance is a feature of many psychometric functions, including temporal discounting with rewarding outcomes [36], [37], [38], and the scale invariance of dread presents a target for future study.

Why dread is a consistent feature of pain related decision-making is unclear. One possibility is that cognitive and emotional mechanisms associated with preparation for pain interfere with other behavioral processes, such as those involved in reward seeking. It is known for example that non-contingent prediction of shock, signaled by a conditioned stimulus, can reduce the vigor of instrumental responding, an effect referred to as conditioned suppression [26]. Dread, as the prospective sum of anticipated punishment, may therefore signal the likely degree of behavioral suppression during the delay. Another possibility is that dread represents a form of ‘stimulus substitution’ – the observation that cues associated with the prediction of aversive events evoke some of the core properties of the aversive events they predict themselves [12], [39]. This can be viewed as a form of aversive impulsivity – assumed to be a maladaptive inheritance of decision-making dispositions that are shaped by earlier evolutionary niches. An alternative explanation would be that people have an increasing uncertainty with time that they can engage in an adequate physical or psychological response to deal with pain. Further research is required to uncover the constitutive mechanisms of dread, which is of importance for clinicians and health policy makers, since knowledge about the shape of pain value functions and their modulation by framing may be useful when presenting options regarding potentially painful investigations and treatments.

## Methods

### Ethics statement

The research received approval from the National Health Service National Research Ethics Service, Central London Research Ethics Committee 3 (Ethics number 08/H0716/6, Amendment AM1). All participants gave informed consent before taking part in the study.

### Experiment 1

#### Participants

Thirty-five participants (18 females) took part in the experiment. Participants were recruited by advertisement on the website of the University College London Psychology Subject Pool. All experiments were carried out at the Wellcome Trust Centre for Neuroimaging, University College London and each session lasted around 2 hours. All participants gave full informed consent prior to the experiment. Participants were briefed that they would be making choices between different numbers of moderately painful electric shocks that would be delivered at different points in time.

#### Procedure and design

In each of 2 sessions participants made 95 choices between two options involving between 3 to 12 moderately painful electric shocks, delivered at between 4 to 51 trials in the future. All choices were genuine, with shock delivered faithfully according to subjects' choices.

The painful shocks occurred within a 5 second stimulus train, and the intensity of each discrete shock did not vary. Since the duration of the stimulus was constant, increasing number of shocks was equivalent to an increasing shock rate. The number of shocks within the stimulus train followed a Poisson distribution with uniform probability of receiving a shock at each sampled time interval. Participants were briefed on the probabilistic nature of the outcomes and were informed of the number of shocks they could on average expect to receive for a particular outcome, that is, the mean of the distribution, where it was assumed that participants made their choices based on this number. This was done purely to embed the experiment into a more naturalistic context, and hence elicit more considered choices.

After providing consent, participants underwent a standardized thresholding procedure [40], [41]. The aim of this procedure was to control for between participant variations in pain perception, so that the maximum shock rate used during the experiment corresponded to an approximately equivalent subjective level of discomfort for each participant. We aimed to set a current level such that the five second stimulus at the maximum shock rate (2.8 shocks/s) was rated as moderately severe pain by each participant. To achieve this we used an expected shock rate of 2.8 shocks/s whilst varying current amplitude. Participants provided a visual analog pain rating for each stimulus train on a continuous scale from 0 (not painful) to 10 (intolerable pain). Current level was increased in small increments until the participant rated the stimulus as 6 out of 10. The staircase procedure was then repeated, allowing participants to adapt to initial anxiety about the shocks. This procedure determined a single current level corresponding to moderately severe pain for each participant. At the end of the experiment we also verified that increasing the mean shock rate within the range used for the experiment corresponded to monotonic increases in rated aversiveness, by asking participants to provide visual analog ratings of the unpleasantness of stimulus trains with different mean shock rates but constant current amplitude. Shock rate was increased in increments of 2 shocks/5s, starting from the baseline mean rate of 2 shocks/5s up to the maximum rate of 14 shocks/5s at a constant current level equal to that used during the choice phase. This was followed by a symmetrical decreasing staircase in which shock rate was decreased by the same increment, thus controlling for adaptation effects. 2 out of the 35 participants were excluded from the analysis on the basis of these ratings, since at the end of the experiment they rated the maximum shock rate as below 4/10 (which corresponded to “mild pain” on the visual analog rating scale), suggesting that significant adaptation had occurred over the course of the experiment.

Prior to the intertemporal choice phase, participants were briefed with on-screen instructions that embedded the task in a naturalistic health-related scenario. We collected intertemporal choice data in two blocks, the order of which was counterbalanced across participants: a block in which outcomes were framed as an increase in shock rate, referred to as the *pain* frame and an otherwise identical experimental block in which outcomes were framed as a decrease in shock rate, referred to as the *relief* frame (see Text S1 for a full description of the information given to participants). Prior to making choices participants received six samples of five second stimulus trains at two different shock rates, corresponding to the minimum and maximum rates used during the experiment, of 2 shocks/5s (0.4 shocks/s) and 14 shocks/5s (2.8 shocks/s). Choice blocks proceeded according to a trial-based design in which the unit of time was a single trial and participants' choices determined outcomes on future trials. The sequence of events across a series of trials is shown in Figure 1.

On each trial the default outcome was a five-second shock train with mean 2 shocks/5s (0.4 shocks/s), which was referred to as a “Baseline Episode”. Participants' choices determined outcomes with higher shock rates, referred to as “Severe Episodes”. Participants were informed that their choices would not change the total number of pain episodes, only their timing and severity. When presenting choice options, shock rate was expressed in the *pain* frame as the expected number of extra shocks per five seconds above the baseline rate and in the *relief* frame as the expected number of shocks to be relieved per five seconds from an expected maximum rate. The timing of outcomes was expressed as the number of trials in the future. Since we made no *a priori* assumptions about the direction of participants' time preference, there were an equal number of choices in which the delayed outcome had a higher expected shock rate as choices in which the sooner outcome had a higher expected shock rate. The presentation of outcome options was counterbalanced and randomized such that sooner outcome options appeared on the left-hand side of the screen on half of trials, and on the right-hand side in the other half of trials.

There were two types of trial: Choice Trials and No Choice Trials. The latter are necessary to absorb the outcomes of all the Choice Trials, such that all choices faithfully and precisely led to their outcomes. In each run there were 95 Choice Trials and an approximately equal number of No Choice Trials. On Choice Trials participants were first presented with a choice between two options for a Severe Episode, where each detailed its timing and expected shock rate. After a choice had been made, there followed the painful episode (five-second outcome stimulus) for that trial, whose shock rate was determined by previous choices. On No Choice Trials participants were presented with a screen saying “No Choice This Time”, which was displayed for a constant delay of 1s, and followed directly by the painful episode for that trial. Choices and outcomes were interleaved: for example if a participant chose on trial one to receive “a Severe Episode with nine extra shocks, five trials in the future”, then following their choice on trial six they would experience an outcome with a mean shock rate of nine shocks above the baseline. The outcome for trial one would then be a Baseline Episode, as was the case for all trials not referenced by a previous choice. Prior to each experimental run we generated a novel trial order using a random permutation that was bounded such that no two Choice Trials referred to an outcome on the same trial, and participants were informed of this fact. Although Choice Trials and No Choice Trials were randomly interspersed, the frequency of the latter necessarily increased towards the end of the experimental run, in order to ensure that choice delays did not extend beyond the end of the experiment.

Shocks were delivered using a Digitimer (Letchworth Garden City, England) DS7 constant current stimulator through silver chloride surface electrodes placed approximately 3 cm apart on the dorsum of the left hand. Each individual shock consisted of a single 200 µs square-wave pulse. Throughout the experiment the participant sat in front of a computer monitor; where trials were presented on-screen, and decisions were indicated using two keys on the keyboard. The software package COGENT 2000 (University College London) was used for stimulus presentation and response acquisition. At the end of the session participants were fully debriefed and given an opportunity to make any comments.

#### Model fitting procedures

To estimate the likelihood of each of the models, we used assumed a standard probabilistic model of action selection in which the probability of choosing option *i* over option *j* depends on a softmax function with the following form:
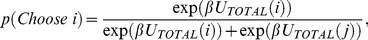
(12)where 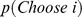 is the probability of choosing option 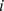 over option 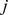, and 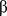 is the inverse temperature parameter. Higher values of 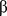 lead to behavior becoming more deterministic for choosing the option with higher utility. The above can be rearranged to the following form,
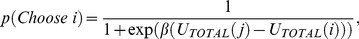
(13)which demonstrates that the probability of choosing an option in this case depends on the difference between the utility of the two options. Simplex optimization was performed using the Matlab (Mathworks, MA, USA) *fminsearch* optimization tool (Nelder-Mead search algorithm [42]), with the addition of bound constraints by transformation, to estimate the parameters for each model in order to maximize log likelihood of the model parameters (by minimizing the negative log likelihood) given the observed binary choices for each subject. The optimizer was called within a random multi-started overlay (RMsearch), with 100 starting points selected from a uniform distribution between the parameter bounds, in order to reduce convergence on local minima. In addition, for each subject 1000 iterations of the optimization were performed, and the maximum likelihood estimate across all iterations was selected. Discount parameters, 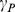 and 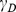, and the dread parameter, 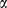, were bounded between 0 and 1. For the Fixed Dread Cost model, the dread cost, *A*, was bounded between 0 and −50. The inverse temperature parameter, 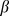, was bounded between 0 and 1000. As an additional safeguard against convergence on local minima, we also performed a grid search for the three best performing models with three-dimensional parameter spaces (Constant Dread, Undiscounted Exponential Dread and the restricted version of Discounted Exponential Dread), evaluating the functions over the entire parameter space, by sampling on a log scale 100 values of each parameter between the bounds. A second grid search was then performed at ten times this resolution in the regions of the maximum likelihood estimates resulting from both the first grid search and the Simplex optimization. Figure S7 plots the results of the second grid search against the Simplex optimization results for each parameter across the three dread-discounting models. Differences in the maximized likelihood between the two search strategies were negligible, and therefore Simplex estimates were retained for the purposes of model comparison. For models with more than three parameters we confirmed that the model-fitting procedure had indeed minimized the negative log-likelihood, by numerically computing the second partial derivatives of the likelihood surface with respect to each parameter in the region of the maximum likelihood estimate.

Model fitting resulted in a set of maximum likelihood parameter estimates for each subject. Model comparison was performed at the group level (fixed effects), by summation of log likelihoods across participants. Selection between models proceeded using the Bayesian Information Criterion (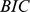) [43], where
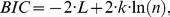
(14)and *L* is the maximized group level log likelihood, *k* is the number of free parameters in the model and *n* the number of independent observations. The 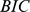 favors models with higher likelihood estimates and penalizes increasing model complexity, where lower values of 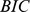 indicate a more favorable model fit. Where appropriate, nested models were compared using likelihood ratio significance tests, where fixed-effects comparisons on the summed group-level likelihoods were of primary interest. For these model comparisons, the total likelihood was summed across all choices regardless of frame (pain or relief).

#### Analysis of framing effects

To examine which model parameters were capable of accounting for choice variability between *pain* and *relief* frames, we fitted the general form Exponential Dread model to data from both frames separately, whilst constraining selected parameters to be the same across both frames (Figure 5C). The fully unrestricted model (All-Framing) had eight parameters, namely: 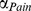, 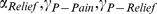, 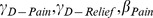 and 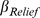. Alternative restricted models were tested in which only a single parameter at time was allowed to vary between frames (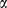-framing, 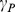-framing, 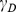-framing and 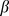-Framing), as well as the fully restricted model in which all three parameters were fixed between frames (No-Framing). 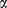-Framing had the following five parameters: 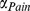, 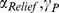, 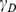 and 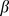. 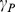-Framing had the following five parameters: 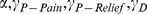 and 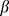. 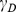-Framing had the following five parameters: 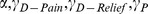 and 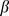. 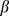-Framing had the following five parameters: 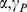, 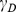 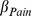, and 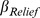. No-Framing had the following four parameters: 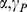, 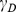 and 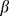. The goodness-of-fit of the alternative restricted models was compared using the summed 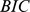 values across both frames, as well as likelihood ratio tests between the two leading models.

### Experiment 2

#### Participants

30 participants took part in the experiment. Participants were recruited by advertisement on the website of the University College London Psychology Subject Pool. Choices were administered online using Qualtrics software (Qualtrics.com; Provo, UT). The study procedure was approved by the joint ethics committee of The National Hospital for Neurology and Neurosurgery and the Institute of Neurology (UCL).

#### Procedure and data analysis

Participants were introduced to the following scenario:

*You are due to have a routine dental appointment. The appointment will last for 15 minutes. The appointment is non-urgent but must be booked now to occur sometime in the next year. Importantly the timing will not affect your dental health: having the appointment sooner will provide no added health benefits. However the experience will be very uncomfortable, and at times painful. Exactly how painful will depend on when you choose to have the appointment. The pain will only last for the length of the appointment: you do not experience dental pain at any other time. The dental surgery is very close to where you live, so you will be able to attend an appointment almost immediately if you choose to. As far as your diary is concerned, any of the appointment times are equally possible for you.*

Participants were offered binary choices between different timings for the dental appointment, in units of days. The sooner appointment was always designated as occurring “today”. The later appointment occurred at delays of 1, 5, 13, 32, 89 or 237 days. In each case participants were told how painful they could expect the appointment to be on a scale of 0% to 100%, where 100% represents the worse imaginable dental pain. The outcome magnitudes were 60, 55, 51 46, 37 and 16% dental pain. At each possible delay, each possible magnitude was paired with an outcome of 60% dental pain. Options were counterbalanced, so that there were an equal number of choices in which the larger magnitude pain was the delayed option as choices in which the smaller magnitude pain was the delayed option. So that the timings appeared plausible, participants were asked to imagine that it was currently a weekday morning. Data analysis followed the same methodology as described above for Experiment 1.

## Supporting Information

Figure S1
**Temporal value functions predicted by alternative models.** For each panel the value of a shock is plotted against increasing delay to its delivery. The value of immediate shock is given by the intersection of the curves with the vertical axis; for purposes of clarity the scales of the vertical axes are arbitrary and differ between the plots. Parameters of the function are displayed next to each. The top left panel depicts simple exponential discounting with positive rate, with the result that the prospective utility of shock becomes less negative the further it is delayed into the future. The top right panel depicts exponential discounting with negative rate, a model which we reject *a priori* due to its implausible prediction that very small values of distantly delayed shock ought to be equivalent to severe immediate shock. The middle left panel depicts a model in which all values of delayed shock carry a fixed subtractive cost, A (here set arbitrarily to a value of 5), with the discount factor 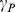 set to 0.95 (see text). The remaining panels depict dread-discounting models as labelled Constant Dread, Undiscounted Exponential Dread (denoted by the prefix U) and Restricted Discounted Exponential Dread (denoted by the prefix D). For the Exponential Dread models depicted, the discount rate used to determine dread is equal to the discount rate applied to consumption of shock, 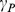.(TIF)

Figure S2
**Parameterization of the Constant Dread model.** A range of temporal value functions predictable by a Constant Dread model, at different values of the two free parameters 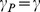 and 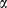. **A**: Effects of increasing 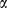 (left to right) at lower (top row) and higher (second row) values of 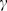. It is evident that at small values of 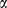 the model approaches positive exponential discounting (top left panel). Simple exponential discounting is produced when 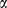 = 0. At positive 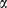 the functions approach linear decreases, where a determines the slope. B: Effects of decreasing 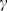 (left to right) at lower (top row) and higher (second row) values of 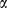.(TIF)

Figure S3
**Parameterization of the general form Exponential Dread model.** A range of temporal value functions predictable by an Exponential Dread model with separate 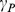 and 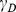, at different values of the three parameters 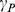, 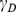 and 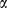. The model allows for points of maximal aversion at intermediate values of delay. **A:** Effects of increasing 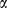 (left to right) at lower (top row) and higher (second row) values of 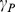 with a high value of 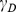. It is evident that at small values of 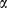 the model approaches positive exponential discounting (top left panel). Simple exponential discounting is produced when 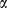 = 0. **B:** Effects of decreasing 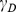 (i.e. increasing the discounting of dread; left to right) at lower (top row) and higher (second row) values of 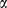.(TIF)

Figure S4
**Parameterization of the Undiscounted Exponential Dread model.** A range of temporal value functions predictable by an Exponential Dread model with 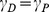, where dread itself is not subject to discounting, at different values of the two free parameters 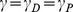, and 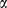. **A**: Effects of increasing 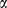 (left to right) at lower (top row) and higher (second row) values of 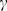. It is evident that at small values of 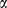 the model approaches positive exponential discounting (top left panel). Simple exponential discounting is produced when 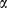 = 0. At positive 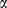 aversiveness (negative value) increases at a decreasing rate with delay, where both 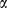 and 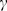 influence the asymptotic boundary. **B**: Effects of decreasing γ (left to right) at lower (top row) and higher (second row) values of 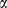.(TIF)

Figure S5
**Parameterization of the Restricted Discounted Exponential Dread model.** A range of temporal value functions predictable by an Exponential Dread model with 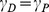, where dread itself is temporally discounted, at different values of the two free parameters γ and 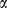. The model allows for points of maximal aversion at intermediate values of delay. **A**: Effects of increasing 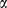 (left to right) at lower (top row) and higher (second row) values of γ. It is evident that at small values of 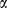 the model approaches positive exponential discounting (top left panel). Simple exponential discounting is produced when 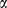 = 0. **B**: Effects of decreasing γ (left to right) at lower (top row) and higher (second row) values of 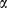.(TIF)

Figure S6 **Utility functions derived from subjective pain ratings.** Visual Analogue Scale (VAS) pain ratings as a function of stimulus shock rate for the 25 participants included in the modeling analysis. VAS ratings were made on a scale ranging from 0 (no pain) to 10 (intolerable pain). Ratings scores were fitted with a 3-parameter concave Weibull function, using least squares minimisation, indicated by the dashed lines on each plot. These functions were then entered as negative utility functions for pain as a function of shock rate in a second modeling analysis of the intertemporal choice data.
(TIF)

Figure S7 **Grid search of parameter space.** Grid search was performed over the entire parameter space of the three best fitting dread-discounting models with up to three dimensional parameter spaces (Constant Dread, Undiscounted Exponential Dread and the restricted version of Discounted Exponential Dread) in order to verify that the random multi-started Simplex optimization procedure successfully avoided local minima in the likelihood surface. Maximum likelihood estimates resulting from grid search of the three parameters are plotted against the estimates resulting from Simplex optimization on a log scale. Outliers, representing cases in which the Simplex routine encountered local minima, are few in number, and in each case in the maximised log likelihood between the two search routines are negligible (they do not change the results of model comparison).
(TIF)

Figure S8
**Fitted temporal value functions: Negative time preference sub-group.** Empirical temporal value functions predicted by the 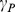-framing version of the general form Exponential Dread model for individuals categorized behaviorally as having consistent negative time preference: subject numbers are indicated above each plot (corresponding to Table S1). Asterisks indicate subjects who showed significant framing effects in the expected direction at the behavioral level (Fisher exact test, *p*<0.05). Note variable scaling of the vertical axes for some individuals, a function of variable softmax temperatures. The blue line represents the value function for the *relief* frame, the red line the value function for the *pain* frame. In each case, where significant behavioral framing effects occurred, this was captured by the model. It can be appreciated that for the majority of subjects the value function for the *pain* frame appears to lie below that in the *relief* frame, consistent with higher dread.(TIF)

Figure S9
**Fitted temporal value functions:** ***zero*** **,** ***positive*** **and** ***reversing*** **time preference sub-groups.** Empirical temporal value functions predicted by the 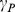-framing version of the general form Exponential Dread model for individuals categorized behaviorally as having either zero time preference (A), consistent positive time preference (B), or negative time preference followed by positive time preference (C). Subject numbers are indicated above each plot (corresponding to Table S1). The asterisks indicate participants with significant framing effects at the behavioural level for individual participants (Fisher exact test, *p*<0.05), the prefix “rev” indicates a single subject (7) who showed a significant framing effect in the direction opposite to that expected. Note variable scaling of the vertical axes for some individuals, a function of variable softmax temperatures. The blue line represents the value function for the relief frame, the red line the value function for the pain frame.(TIF)

Table S1 **Choice frequency: Experiment 1.** The table outlines the percentage of choices in which the sooner painful episode was chosen for each participant, on both *pain* and *relief* frames, as well as the percentage difference between the two frames (percentage sooner choice on the pain frame minus the percentage sooner choice on the relief frame). The latter indicates the size of the framing effect; positively signed values of this difference indicate that the participant choose sooner pain more frequently in the *pain* frame, indicating a framing effect in the expected direction. The final two columns show the *p*-value and hypothesis test of a Fisher exact test on the percentage choices in each frame; a 1 in the final column indicates a significant framing effect in the expected direction at an individual level. Table S1 outlines the overall frequencies of choosing sooner pain in both pain and relief frames in Experiment 1. The eight participants listed in gray chose sooner pain 100% of the time in at least one of the two frames, rendering their data unsuitable for model-based analysis. 10 out of 33 participants displayed significant framing effects in the expected direction at an individual level (Fisher exact test *p*<0.05).
(DOC)

Table S2 **Model parameters for the general form Exponential Dread model with γ_P_-framing: Experiment 1.** Maximum likelihood parameter estimates are listed for the five parameters of the general form Exponential Dread model with γ_P_-framing for each participant included in the modeling analysis for Experiment 1. Participant numbers correspond to those in Table S1.
(DOC)

Text S1 **Information given to participants in Experiment 1.** Details the instructions in Experiment 1 which embed the task within a health-related scenario and distinguish the two frames.
(DOC)
