# Supplementary figures and images for: Dread and the Disvalue of Future Pain
Source: PLoS Comput Biol. 2013 Nov 21;9(11):e1003335. doi: 10.1371/journal.pcbi.1003335 (PMC3836706; doi:10.1371/journal.pcbi.1003335)

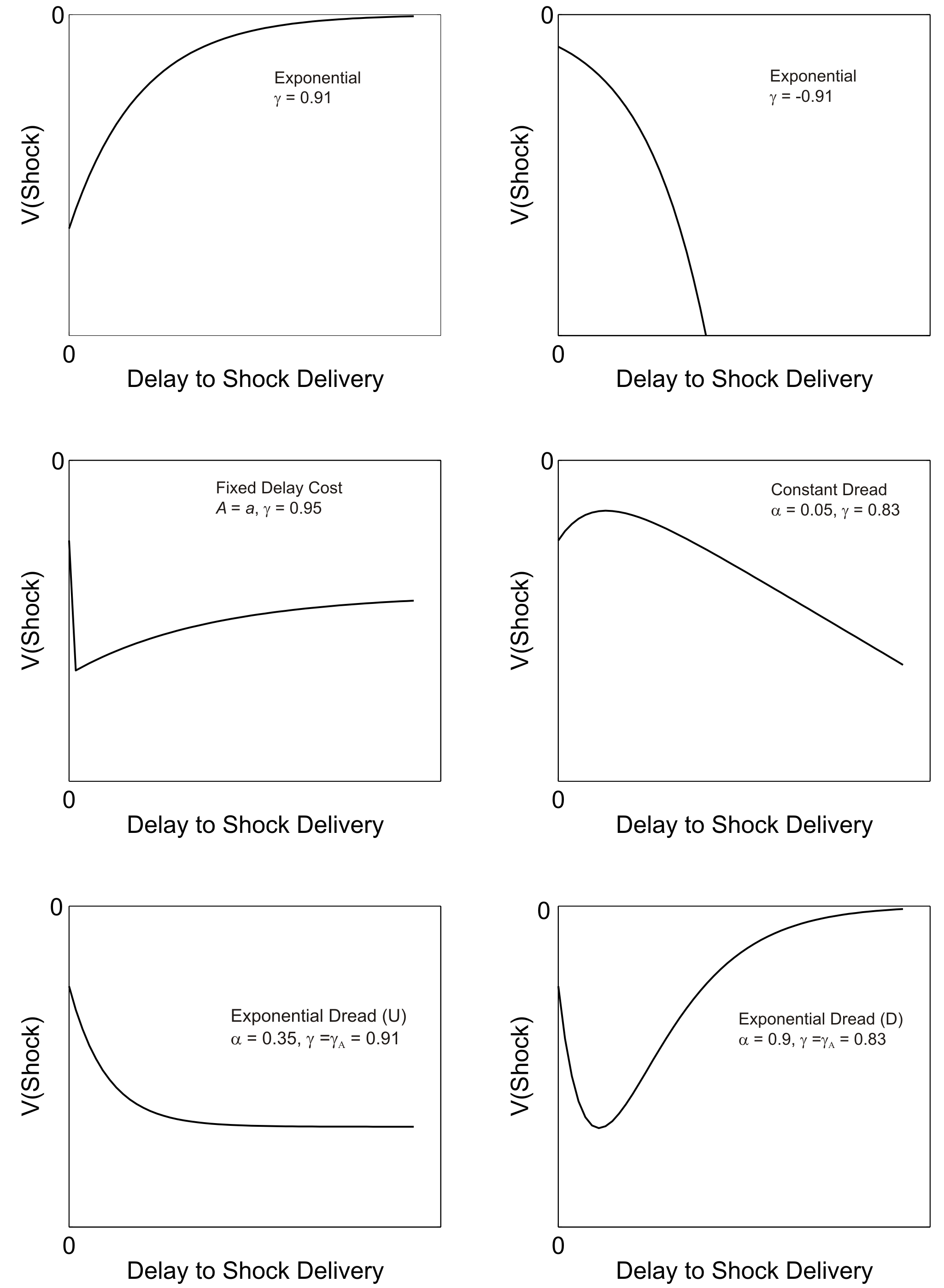

Supplement: Figure S1 — Temporal value functions predicted by alternative models. For each panel the value of a shock is plotted against increasing delay to its delivery. The value of immediate shock is given by the intersection of the curves with the vertical axis; for purposes of clarity the scales of the vertical axes are arbitrary and differ between the plots. Parameters of the function are displayed next to each. The top left panel depicts simple exponential discounting with positive rate, with the result that the prospective utility of shock becomes less negative the further it is delayed into the future. The top right panel depicts exponential discounting with negative rate, a model which we reject a priori due to its implausible prediction that very small values of distantly delayed shock ought to be equivalent to severe immediate shock. The middle left panel depicts a model in which all values of delayed shock carry a fixed subtractive cost, A (here set arbitrarily to a value of 5), with the discount factor set to 0.95 (see text). The remaining panels depict dread-discounting models as labelled Constant Dread, Undiscounted Exponential Dread (denoted by the prefix U) and Restricted Discounted Exponential Dread (denoted by the prefix D). For the Exponential Dread models depicted, the discount rate used to determine dread is equal to the discount rate applied to consumption of shock, . (TIF) [file pcbi.1003335.s001.tif]

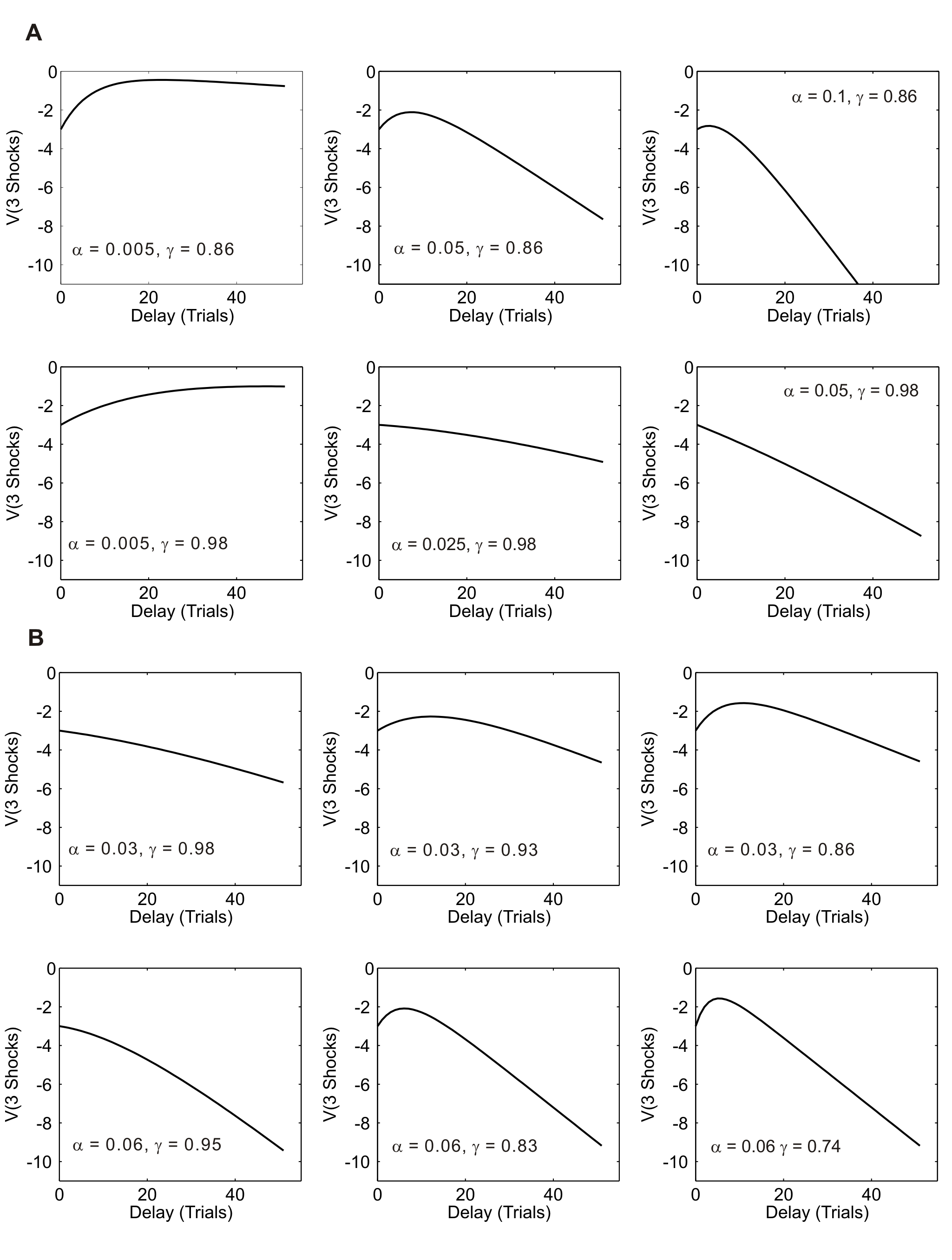

Supplement: Figure S2 — Parameterization of the Constant Dread model. A range of temporal value functions predictable by a Constant Dread model, at different values of the two free parameters and . A: Effects of increasing (left to right) at lower (top row) and higher (second row) values of . It is evident that at small values of the model approaches positive exponential discounting (top left panel). Simple exponential discounting is produced when = 0. At positive the functions approach linear decreases, where a determines the slope. B: Effects of decreasing (left to right) at lower (top row) and higher (second row) values of . (TIF) [file pcbi.1003335.s002.tif]

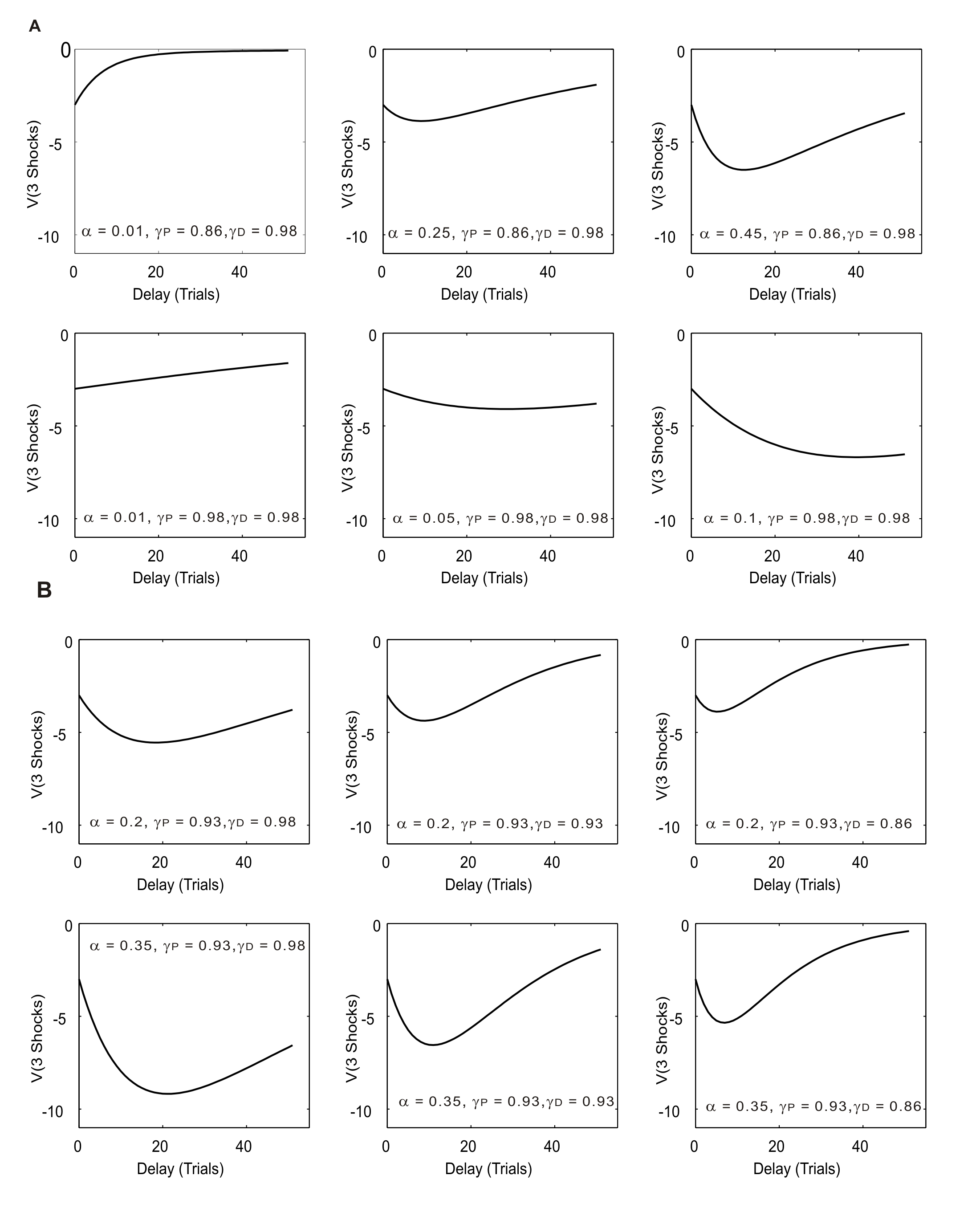

Supplement: Figure S3 — Parameterization of the general form Exponential Dread model. A range of temporal value functions predictable by an Exponential Dread model with separate and , at different values of the three parameters , and . The model allows for points of maximal aversion at intermediate values of delay. A: Effects of increasing (left to right) at lower (top row) and higher (second row) values of with a high value of . It is evident that at small values of the model approaches positive exponential discounting (top left panel). Simple exponential discounting is produced when = 0. B: Effects of decreasing (i.e. increasing the discounting of dread; left to right) at lower (top row) and higher (second row) values of . (TIF) [file pcbi.1003335.s003.tif]

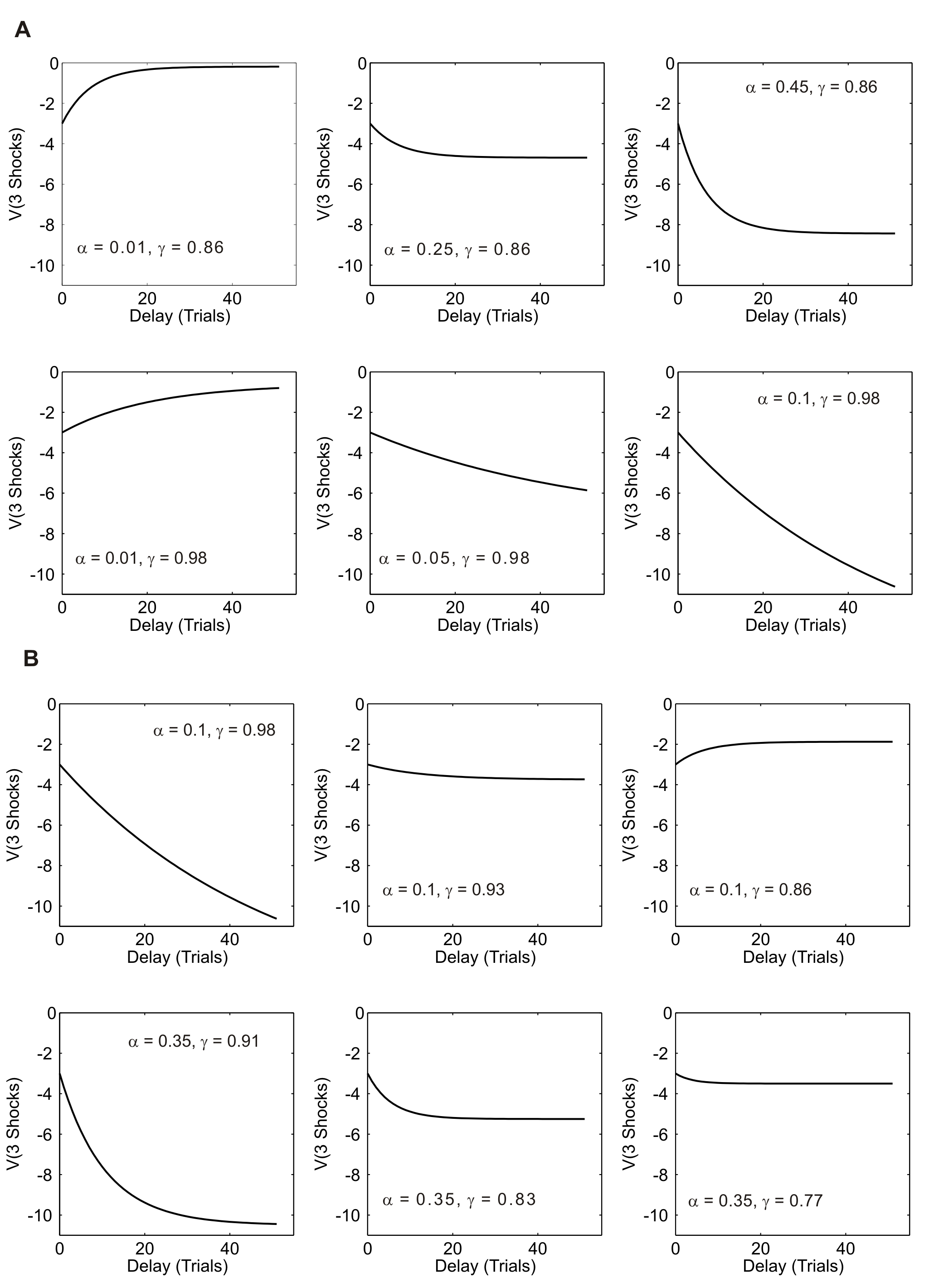

Supplement: Figure S4 — Parameterization of the Undiscounted Exponential Dread model. A range of temporal value functions predictable by an Exponential Dread model with , where dread itself is not subject to discounting, at different values of the two free parameters , and . A: Effects of increasing (left to right) at lower (top row) and higher (second row) values of . It is evident that at small values of the model approaches positive exponential discounting (top left panel). Simple exponential discounting is produced when = 0. At positive aversiveness (negative value) increases at a decreasing rate with delay, where both and influence the asymptotic boundary. B: Effects of decreasing γ (left to right) at lower (top row) and higher (second row) values of . (TIF) [file pcbi.1003335.s004.tif]

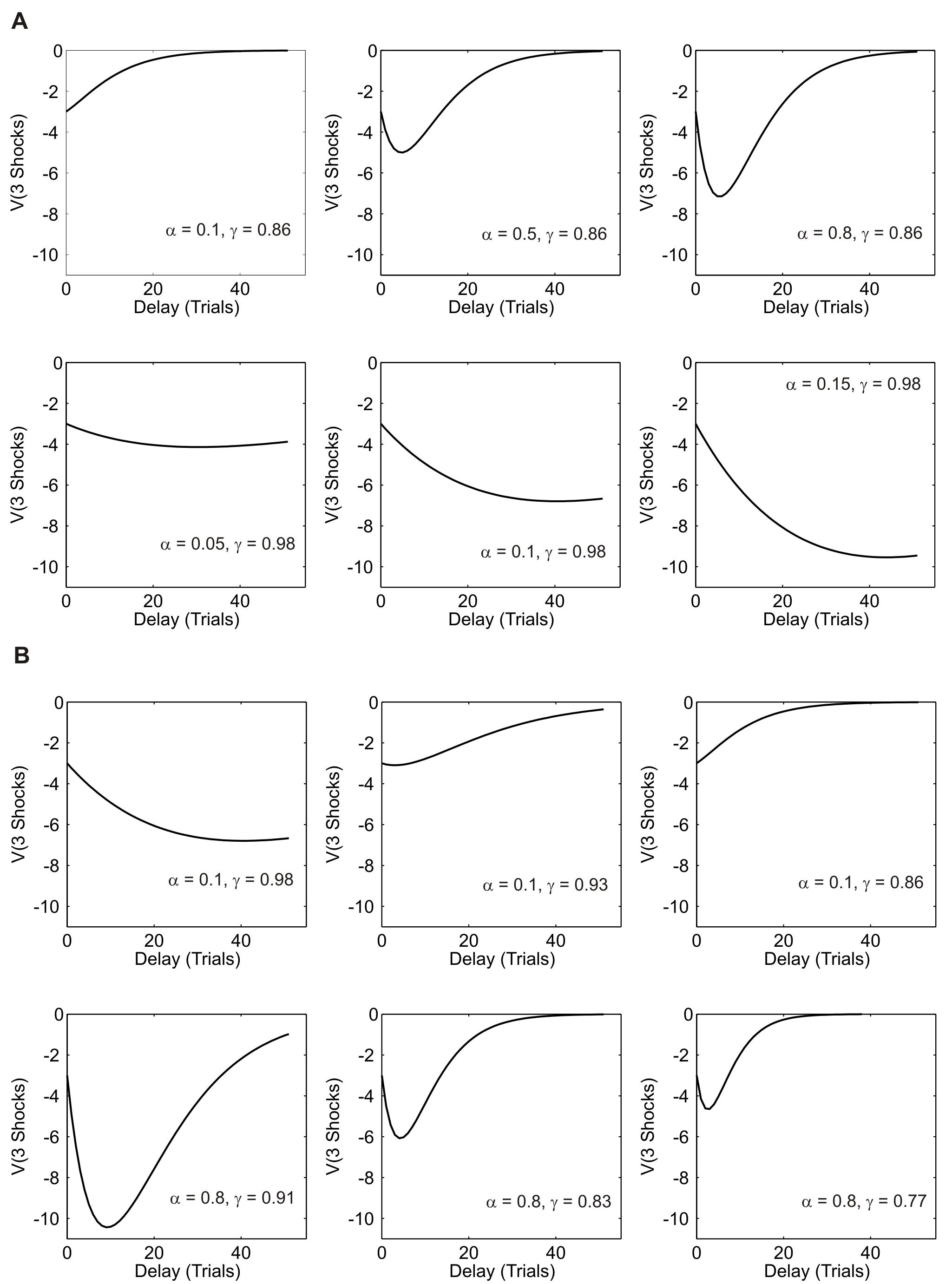

Supplement: Figure S5 — Parameterization of the Restricted Discounted Exponential Dread model. A range of temporal value functions predictable by an Exponential Dread model with , where dread itself is temporally discounted, at different values of the two free parameters γ and . The model allows for points of maximal aversion at intermediate values of delay. A: Effects of increasing (left to right) at lower (top row) and higher (second row) values of γ. It is evident that at small values of the model approaches positive exponential discounting (top left panel). Simple exponential discounting is produced when = 0. B: Effects of decreasing γ (left to right) at lower (top row) and higher (second row) values of . (TIF) [file pcbi.1003335.s005.tif]

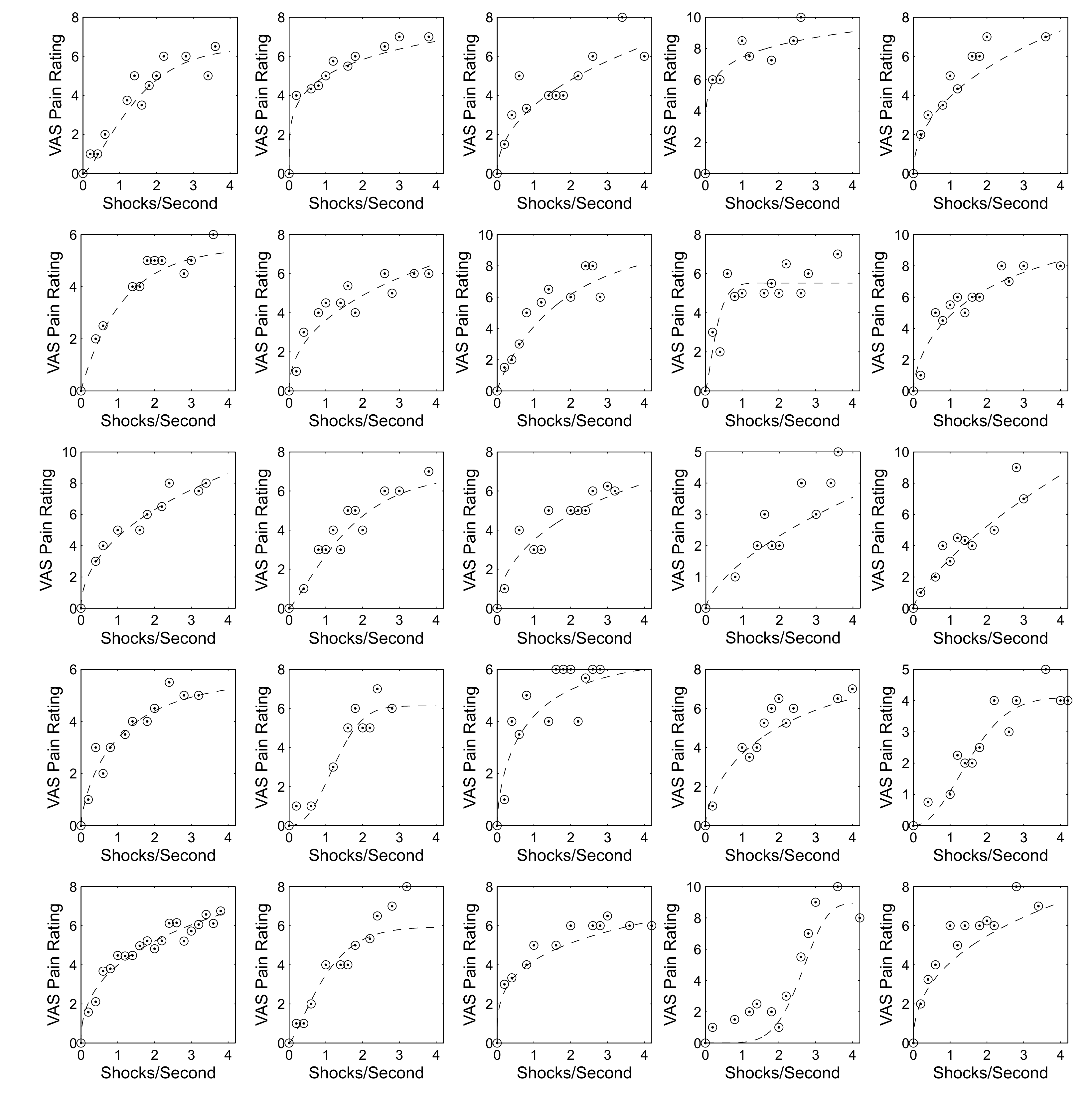

Supplement: Figure S6 — Utility functions derived from subjective pain ratings. Visual Analogue Scale (VAS) pain ratings as a function of stimulus shock rate for the 25 participants included in the modeling analysis. VAS ratings were made on a scale ranging from 0 (no pain) to 10 (intolerable pain). Ratings scores were fitted with a 3-parameter concave Weibull function, using least squares minimisation, indicated by the dashed lines on each plot. These functions were then entered as negative utility functions for pain as a function of shock rate in a second modeling analysis of the intertemporal choice data. (TIF) [file pcbi.1003335.s006.tif]

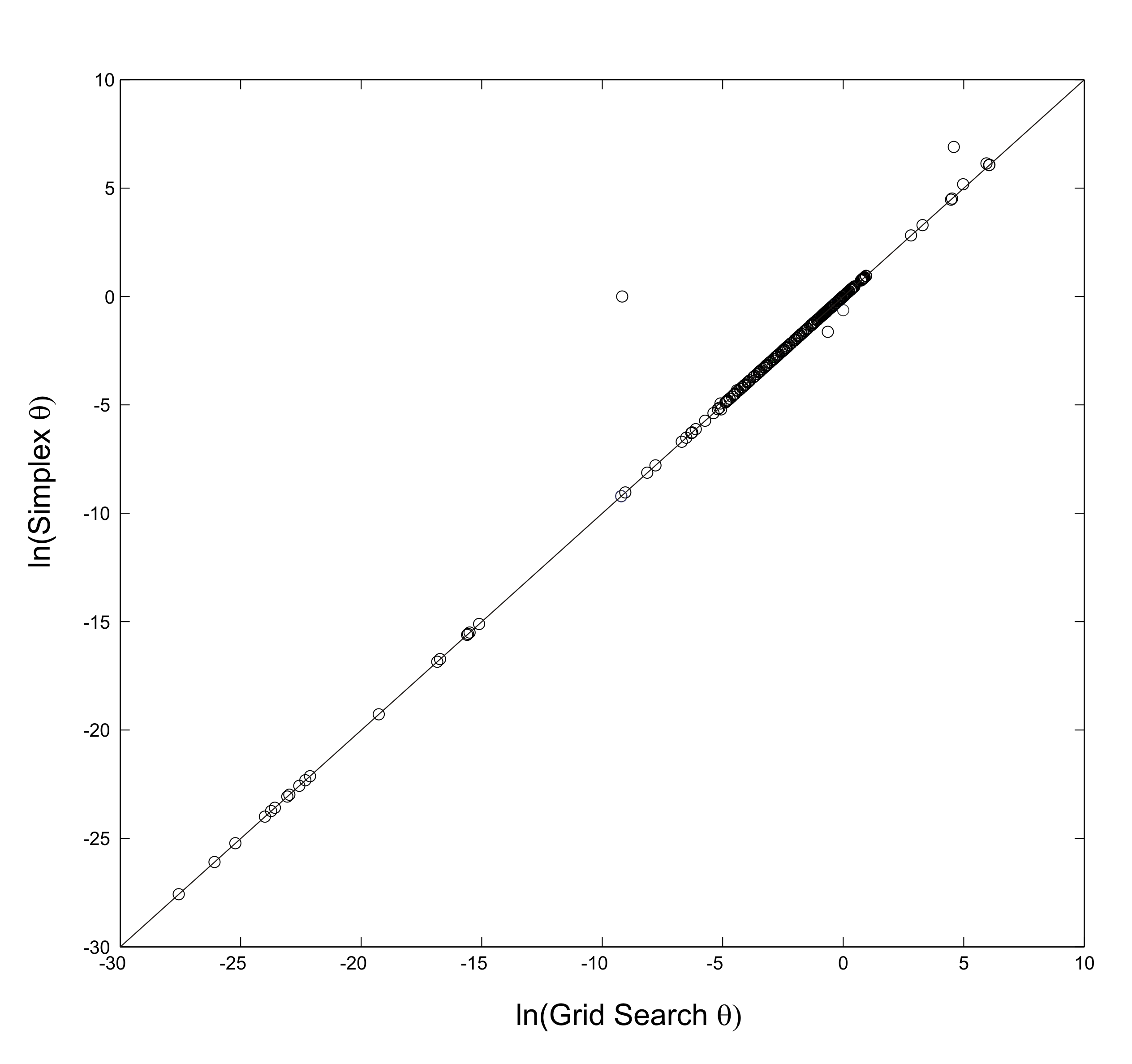

Supplement: Figure S7 — Grid search of parameter space. Grid search was performed over the entire parameter space of the three best fitting dread-discounting models with up to three dimensional parameter spaces (Constant Dread, Undiscounted Exponential Dread and the restricted version of Discounted Exponential Dread) in order to verify that the random multi-started Simplex optimization procedure successfully avoided local minima in the likelihood surface. Maximum likelihood estimates resulting from grid search of the three parameters are plotted against the estimates resulting from Simplex optimization on a log scale. Outliers, representing cases in which the Simplex routine encountered local minima, are few in number, and in each case in the maximised log likelihood between the two search routines are negligible (they do not change the results of model comparison). (TIF) [file pcbi.1003335.s007.tif]

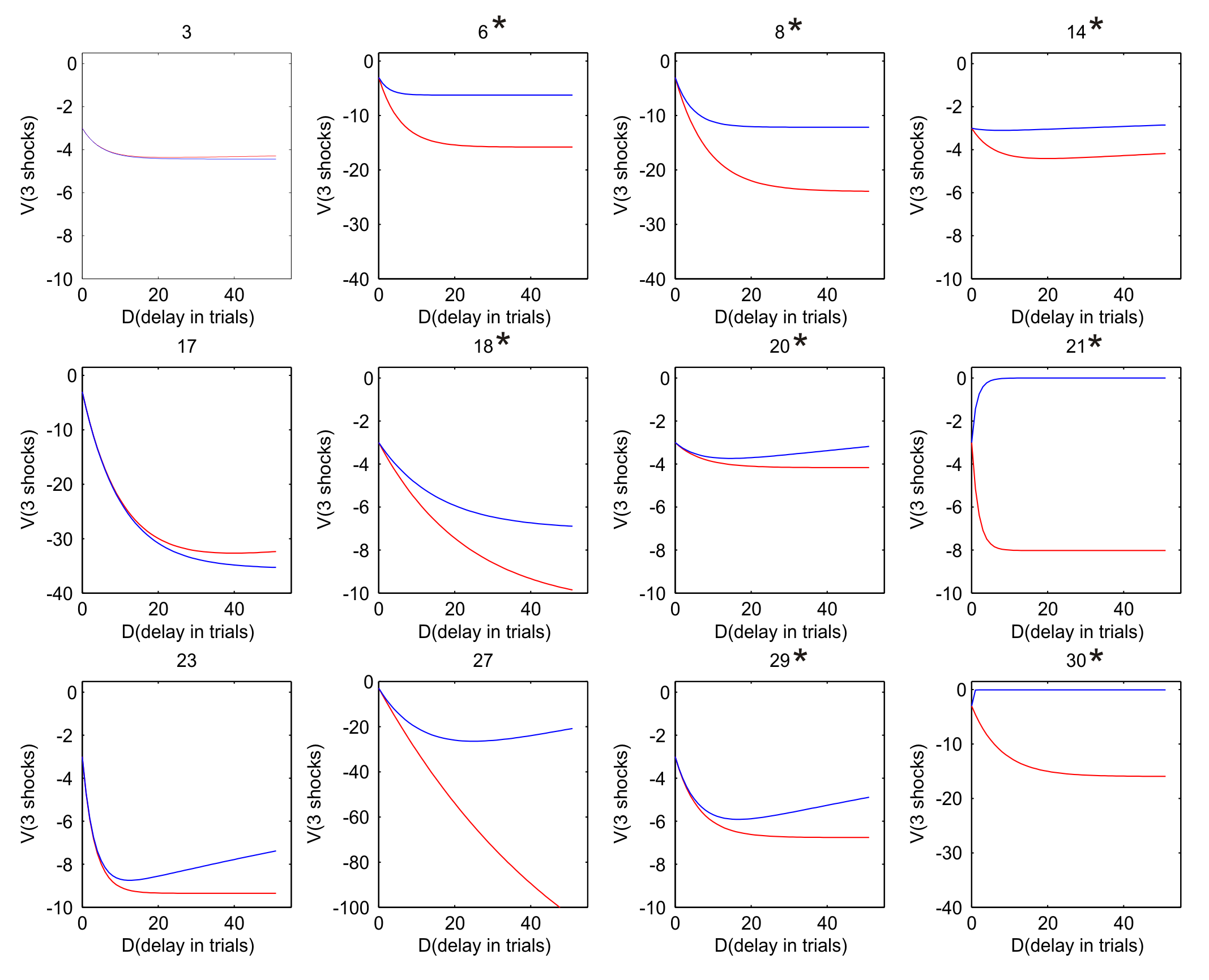

Supplement: Figure S8 — Fitted temporal value functions: Negative time preference sub-group. Empirical temporal value functions predicted by the -framing version of the general form Exponential Dread model for individuals categorized behaviorally as having consistent negative time preference: subject numbers are indicated above each plot (corresponding to Table S1). Asterisks indicate subjects who showed significant framing effects in the expected direction at the behavioral level (Fisher exact test, p<0.05). Note variable scaling of the vertical axes for some individuals, a function of variable softmax temperatures. The blue line represents the value function for the relief frame, the red line the value function for the pain frame. In each case, where significant behavioral framing effects occurred, this was captured by the model. It can be appreciated that for the majority of subjects the value function for the pain frame appears to lie below that in the relief frame, consistent with higher dread. (TIF) [file pcbi.1003335.s008.tif]

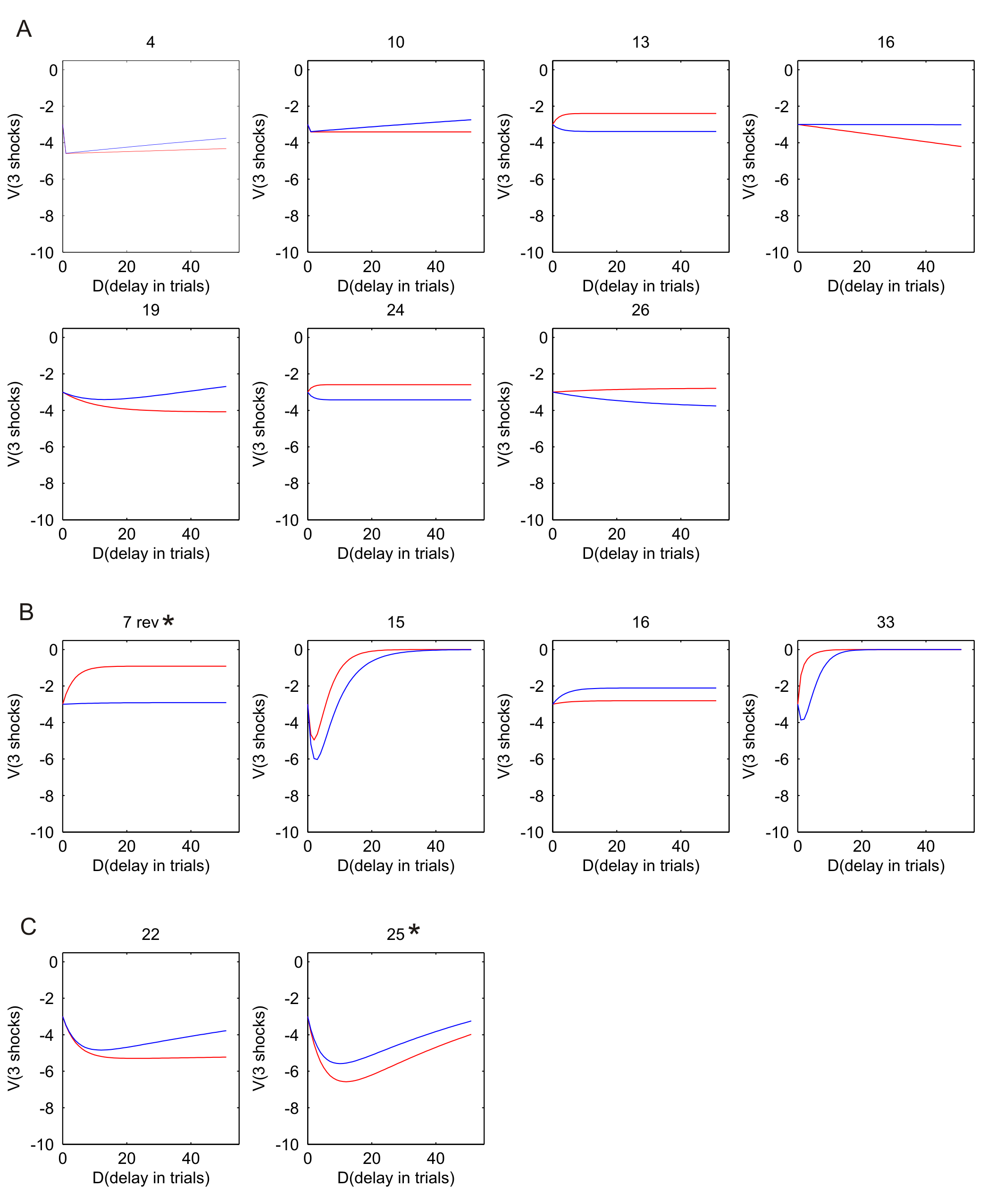

Supplement: Figure S9 — Fitted temporal value functions: zero , positive and reversing time preference sub-groups. Empirical temporal value functions predicted by the -framing version of the general form Exponential Dread model for individuals categorized behaviorally as having either zero time preference (A), consistent positive time preference (B), or negative time preference followed by positive time preference (C). Subject numbers are indicated above each plot (corresponding to Table S1). The asterisks indicate participants with significant framing effects at the behavioural level for individual participants (Fisher exact test, p<0.05), the prefix “rev” indicates a single subject (7) who showed a significant framing effect in the direction opposite to that expected. Note variable scaling of the vertical axes for some individuals, a function of variable softmax temperatures. The blue line represents the value function for the relief frame, the red line the value function for the pain frame. (TIF) [file pcbi.1003335.s009.tif]
