# Supplementary material for: Dread and the Disvalue of Future Pain
Source: PLoS Comput Biol. 2013 Nov 21;9(11):e1003335. doi: 10.1371/journal.pcbi.1003335 (PMC3836706; doi:10.1371/journal.pcbi.1003335)
Supplement: Table S1 — Choice frequency: Experiment 1. The table outlines the percentage of choices in which the sooner painful episode was chosen for each participant, on both pain and relief frames, as well as the percentage difference between the two frames (percentage sooner choice on the pain frame minus the percentage sooner choice on the relief frame). The latter indicates the size of the framing effect; positively signed values of this difference indicate that the participant choose sooner pain more frequently in the pain frame, indicating a framing effect in the expected direction. The final two columns show the p-value and hypothesis test of a Fisher exact test on the percentage choices in each frame; a 1 in the final column indicates a significant framing effect in the expected direction at an individual level. Table S1 outlines the overall frequencies of choosing sooner pain in both pain and relief frames in Experiment 1. The eight participants listed in gray chose sooner pain 100% of the time in at least one of the two frames, rendering their data unsuitable for model-based analysis. 10 out of 33 participants displayed significant framing effects in the expected direction at an individual level (Fisher exact test p<0.05). (DOC) [file pcbi.1003335.s010.doc]

**Table S1. Choice frequency: Experiment 1**

| Participant | % Sooner Choice Pain Frame | % Sooner Choice  Relief Frame | % Difference in  Sooner Choice  (Framing  Effect) | Fisher Exact  One-tailed  *p* value | Significant  Framing  Expected  Direction |
| --- | --- | --- | --- | --- | --- |
| 1 | 96.8 | 100.0 | -3.2 | 0.123 | 0 |
| 2 | 100.0 | 98.9 | 1.1 | 0.500 | 0 |
| 3 | 63.2 | 69.5 | -6.3 | 0.221 | 0 |
| 4 | 53.7 | 48.4 | 5.3 | 0.281 | 0 |
| 5 | 100.0 | 100.0 | 0.0 | 1.000 | 0 |
| 6 | 68.4 | 54.7 | 13.7 | 0.037 | 1 |
| 7 | 29.5 | 44.2 | -14.7 | 0.025 | 0 |
| 8 | 95.8 | 82.1 | 13.7 | 0.002 | 1 |
| 9 | 98.9 | 100.0 | -1.1 | 1.000 | 0 |
| 10 | 52.6 | 50.5 | 2.1 | 0.442 | 0 |
| 11 | 90.5 | 100.0 | -9.5 | 0.002 | 0 |
| 12 | 100.0 | 88.4 | 11.6 | <0.001 | 1 |
| 13 | 42.1 | 53.7 | -11.6 | 0.073 | 0 |
| 14 | 66.3 | 49.5 | 16.8 | 0.014 | 1 |
| 15 | 37.9 | 26.3 | 11.6 | 0.060 | 0 |
| 16 | 57.9 | 52.6 | 5.3 | 0.280 | 0 |
| 17 | 92.6 | 95.8 | -3.2 | 0.268 | 0 |
| 18 | 92.6 | 81.1 | 11.6 | 0.015 | 1 |
| 19 | 63.2 | 52.6 | 10.5 | 0.093 | 0 |
| 20 | 72.6 | 51.6 | 21.1 | 0.002 | 1 |
| 21 | 72.6 | 30.5 | 42.1 | <0.001 | 1 |
| 22 | 72.6 | 65.3 | 7.4 | 0.173 | 0 |
| 23 | 82.1 | 72.6 | 9.5 | 0.082 | 0 |
| 24 | 49.5 | 52.6 | -3.2 | 0.386 | 0 |
| 25 | 69.5 | 53.7 | 15.8 | 0.018 | 1 |
| 26 | 46.3 | 56.8 | -10.5 | 0.096 | 0 |
| 27 | 98.9 | 97.9 | 1.1 | 0.500 | 0 |
| 28 | 47.4 | 34.7 | 12.6 | 0.052 | 0 |
| 29 | 88.4 | 76.8 | 11.6 | 0.027 | 1 |
| 30 | 95.8 | 53.7 | 42.1 | <0.001 | 1 |
| 31 | 97.9 | 100.0 | -2.1 | 0.249 | 0 |
| 32 | 100.0 | 98.9 | 1.1 | 0.500 | 0 |
| 33 | 36.8 | 29.5 | 7.4 | 0.178 | 0 |
