# Supplementary material for: Dread and the Disvalue of Future Pain
Source: PLoS Comput Biol. 2013 Nov 21;9(11):e1003335. doi: 10.1371/journal.pcbi.1003335 (PMC3836706; doi:10.1371/journal.pcbi.1003335)
Supplement: Table S2 — Model parameters for the general form Exponential Dread model with γP-framing: Experiment 1. Maximum likelihood parameter estimates are listed for the five parameters of the general form Exponential Dread model with γP-framing for each participant included in the modeling analysis for Experiment 1. Participant numbers correspond to those in Table S1. (DOC) [file pcbi.1003335.s011.doc]

**Table S2. Model parameters for the general form Exponential Dread Model with γ_P_-framing: Experiment 1**

| Participant | γ_P_ *Pain* | γ_P_ *Relief* | γ_D_ | α | β |
| --- | --- | --- | --- | --- | --- |
| 3 | 0.999 | 1.000 | 0.817 | 0.087 | 0.981 |
| 4 | 0.999 | 0.996 | 0.001 | 0.531 | 0.646 |
| 6 | 0.840 | 0.676 | 1.000 | 1.000 | 0.052 |
| 7 | 0.740 | 0.901 | 1.000 | 0.107 | 0.412 |
| 8 | 0.889 | 0.802 | 1.000 | 1.000 | 0.139 |
| 10 | 1.000 | 0.996 | 0.000 | 0.135 | 3.059 |
| 13 | 0.564 | 0.646 | 1.000 | 0.617 | 1.424 |
| 14 | 0.842 | 0.785 | 0.998 | 0.287 | 0.961 |
| 15 | 0.776 | 0.864 | 0.579 | 1.000 | 0.121 |
| 16 | 1.000 | 0.992 | 1.000 | 0.008 | 1.716 |
| 17 | 0.998 | 1.000 | 0.908 | 1.000 | 0.073 |
| 18 | 0.958 | 0.938 | 1.000 | 0.156 | 0.463 |
| 19 | 1.000 | 0.991 | 0.908 | 0.033 | 1.622 |
| 20 | 1.000 | 0.995 | 0.866 | 0.052 | 0.544 |
| 21 | 1.000 | 0.278 | 0.571 | 0.717 | 0.417 |
| 22 | 0.999 | 0.993 | 0.794 | 0.163 | 0.881 |
| 23 | 1.000 | 0.995 | 0.735 | 0.561 | 0.321 |
| 24 | 0.464 | 0.533 | 1.000 | 1.000 | 3.194 |
| 25 | 0.825 | 0.795 | 0.985 | 0.552 | 0.289 |
| 26 | 0.950 | 0.964 | 1.000 | 0.048 | 1.678 |
| 27 | 1.000 | 0.932 | 0.982 | 1.000 | 0.130 |
| 28 | 0.810 | 0.762 | 1.000 | 0.219 | 0.697 |
| 29 | 1.000 | 0.993 | 0.849 | 0.189 | 0.831 |
| 30 | 0.877 | 0.016 | 1.000 | 0.743 | 0.246 |
| 33 | 0.234 | 0.643 | 0.691 | 1.000 | 0.156 |

*Notes* See Figures S8 and S9 for the resulting temporal value functions for each participant.
