# Supplementary material for: Dread and the Disvalue of Future Pain
Source: PLoS Comput Biol. 2013 Nov 21;9(11):e1003335. doi: 10.1371/journal.pcbi.1003335 (PMC3836706; doi:10.1371/journal.pcbi.1003335)
Supplement: Text S1 — Information given to participants in Experiment 1. Details the instructions in Experiment 1 which embed the task within a health-related scenario and distinguish the two frames. (DOC) [file pcbi.1003335.s012.doc]

# **Text S1**

*Instructions Given to Participants: Experiment 1*

Prior to the intertemporal choice phase of Experiment 1, participants were briefed using the following on-screen instructions that embedded the task in a naturalistic scenario:

*Imagine that you are suffering from pains as a result of damage to the nerves in your arm, which feel as if you are having brief electric shocks delivered to your hand. The pains come in clusters of ‘shocks’, which are roughly evenly spaced in time, with each episode lasting five seconds.*

*Within each episode the shocks come randomly. You experience a usual baseline level of pain, of on average two shocks per episode. All the shocks are of the same intensity.*

Collection of intertemporal choice data followed a trial-based design in which the unit of time was a single trial and participants’ choices determined outcomes on future trials. Participants’ choices determined outcomes with higher shock rates, referred to as “severe episodes”. The description of these episodes differed between the two frames. The description in the pain frame was as follows:

*You are about to have an attack of more severe episodes, lasting about half an hour in total. These severe episodes consist of more shocks than your usual baseline level, sometimes up to around 12 extra shocks per episode. You can choose when you would like to experience each severe episode: for each episode you will be offered two different points in time, and you will be given the choice as to which you would prefer. The severity of the episode will often be different at the two different time points but, to help you make your choice you will be told the average number of extra shocks, relative to your usual baseline, that you could expect to receive at each time.*

The description in the relief frame was as follows:

*You are about to have an attack of more severe episodes, lasting about half an hour in total. These severe episodes consist of 12 more shocks per episode than your usual baseline level. You can choose when you would like to experience each severe episode: for each episode you will be offered two different points in time, and you will be given the choice as to which you would prefer. The severity of the episode will be 12 shocks above the baseline, but you will be offered a painkiller to reduce the severity. The effectiveness of the painkiller might depend on when it is used. To help you to choose you will be told how many shocks you can expect the painkiller to relieve at each time-point.*
